# Differential modulation of trunk muscle activation using thoracic epidural spinal stimulation

**DOI:** 10.1088/1741-2552/adf9a0

**Published:** 2025-08-22

**Authors:** Sydney M Schadan, Alexander G Steele, Amir H Faraji, Albert H Vette, Dimitry G Sayenko

**Affiliations:** 1 Department of Mechanical Engineering, University of Alberta, Donadeo Innovation Centre for Engineering, 9211 116 Street NW, Edmonton, Alberta T6G 1H9, Canada; 2 Center for Neural Systems Restoration, Department of Neurosurgery, Houston Methodist Research Institute, 6550 Fannin Street, Houston, TX 77030, United States of America; 3 Glenrose Rehabilitation Hospital, Alberta Health Services, 10230 111 Avenue NW, Edmonton, Alberta T5G 0B7, Canada; 4 Department of Biomedical Engineering, University of Alberta, Donadeo Innovation Centre for Engineering, 9211 116 Street NW, Edmonton, Alberta T6G 1H9, Canada; 5 Shared senior authorship.

**Keywords:** electromyography, epidural spinal stimulation, evoked motor potentials, human trunk, muscle activity, neuromodulation

## Abstract

**Objective.:**

Epidural spinal stimulation (ESS) has demonstrated promising functional improvements in trunk control following spinal cord injury (SCI). However, previous ESS studies targeting trunk muscle activation have been limited to stimulation over the eleventh thoracic to first lumbar vertebral levels, which may not be optimal based on anatomical evidence regarding trunk muscle innervation. In this light, the objective of this study was to investigate trunk muscle activity in response to ESS at varying stimulation locations above the thoracic spine.

**Approach.:**

An electrode array was implanted above the thoracic spine of 13 participants. ESS-evoked responses in trunk muscles were quantified while stimulation location along the rostrocaudal and mediolateral axes of the spine was systematically manipulated.

**Main results.:**

Ipsilateral ESS between the T6 and T10 vertebrae induced responses in all trunk muscles, resulting in average motor thresholds (MTs) and latencies of abdominal muscles ranging from 1.5 to 2.0 *μ*C and 7.4 to 9.2 ms, respectively; however, stimulation between the T8 and T10 vertebrae demonstrated lower MTs and shorter latencies. Ipsilateral stimulation resulted in 2.4 times greater maximum response amplitudes, 30% lower MTs, and 0.9 ms shorter latencies compared to contralateral stimulation.

**Significance.:**

Our study provides quantitative evidence on the differential effects of ESS amplitude and location on trunk muscle activity while also suggesting that both afferent and efferent pathways contribute to ESS-evoked muscle activation. The results enhance our fundamental understanding of ESS-induced trunk muscle activity and have the potential to guide electrode placement for future therapeutic or restorative applications toward improving trunk control following SCI.

## 1. Introduction

Neuromuscular impairment, such as chronic spinal cord injury (SCI), can affect sensory and motor functions below the site of injury, greatly impacting independence and quality of life. Among other consequences, individuals with cervical or thoracic SCI often face challenges with trunk stability and control [1]. A lack of trunk stability and control not only jeopardizes the ability to sit upright and perform routine reaching tasks needed for daily function, but can also result in severe secondary health complications, such as pressure sores, spasticity, and compromised internal organ function [2]. Considering these consequences, it is not surprising that affected individuals view restoring trunk stability as one of the top priorities in their rehabilitation objectives [3].

Some conventional technologies, including tilted wheelchairs, belts, or braces, aim to passively increase trunk stability following SCI. Alternatively, active technologies have emerged to purposefully engage the neuromuscular system to activate relevant skeletal muscles and enhance mobility and posture. Transcutaneous spinal stimulation (TSS) is one of such approaches, which can activate spinal circuitry through non-invasive current delivered to the dorsal spinal cord [4]. Activation of spinal circuitry projecting to targeted muscles, including Ia afferents synapsing on *α*-motoneurons, interneurons, ascending sensory fibers, and descending motor tracts, enables spinal stimulation to produce targeted motor responses [5-14]. In the domain of upper body function, TSS has demonstrated promising functional outcomes following SCI, including improved trunk control and upright posture [5, 15, 16]. Moreover, TSS in combination with physical therapy has shown immediate and sustained improvements in upper body sensory and motor function in individuals with SCI, demonstrating its therapeutic potential by facilitating neuroplastic changes within spinal networks [17, 18].

TSS can induce muscle responses depending on stimulation location, frequency, intensity, and pulse width [5-14]. The objective quantification of these muscle responses using electromyography (EMG) serves as a valuable tool for selecting stimulation parameters in TSS applications [5, 14, 16]. Recent findings demonstrate that TSS can differentially activate upper [13, 14] and lower limb muscles [19-21], based on the anatomical arrangements of targeted motor pools within the cervical and lumbosacral enlargements, respectively. An optimized stimulation configuration can direct electric fields toward specific subsets of dorsal root entry zones, thereby modulating selected ensembles of motor neuron pools. This approach aids in enhancing specific motor functions with greater precision and objectivity, aligning with the priorities of targeted neurorehabilitation and restorative interventions [22].

Being able to spatially map the spinal motor pools within the thoracic spinal cord projecting to trunk muscles promises to have significant clinical and electrophysiological implications. At the same time, spatial mapping of thoracic motoneurons using TSS poses challenges due to (1) relatively high stimulation intensities required for the electrical current to activate the motoneurons; and (2) the proximity of the stimulation site to the trunk muscles of interest. For the latter, due to the brief latency between the stimulation event and the onset of the evoked motor response, the evoked motor responses captured via EMG are frequently obscured by stimulation artifacts. This poses difficulties not only in evaluating response latency, but also in discerning small evoked responses from substantial artifacts. In this light, epidural spinal stimulation (ESS) can offer a viable approach, or alternative, for mapping the motor pools located in the thoracic spinal cord. In fact, the proximity of the ESS electrodes to the dorsal roots entering the spinal cord results in significantly smaller electrical current requirements for activating the Ia afferents compared to TSS. Consequently, this also mitigates the issue of large stimulation artifacts obscuring the TSS evoked responses. Importantly, findings from ESS studies are transferable to and suggest similar outcomes for TSS applications [8, 20, 23, 24].

In the context of trunk control and stability, ESS has been used to promote function of trunk muscles [25-27]. Such studies have been limited to stimulation locations within the lower thoracic (T11) to upper lumbosacral (L1) vertebral levels, although Rowald *et al* suggested stimulating additional thoracic dorsal roots for improved trunk muscle activation and postural stability [27]. Furthermore, individuals with lower thoracic SCI demonstrate better dynamic sitting stability than those with high thoracic SCI, implying that trunk muscle innervation for trunk stability is also occurring in the higher thoracic region [28]. This is in line with current neuroanatomical knowledge of the thoracic innervation of several trunk muscles relevant for trunk stability and control [29, 30], e.g. the rectus abdominis (RA), internal obliques (IO), and external obliques (EO).

Based on the considerations above, the objective of this study was to systematically investigate the relative recruitment selectivity of different motor pools projecting to trunk muscles in individuals with no spinal cord pathology, using ESS along the rostrocaudal and mediolateral axes of the thoracic spinal cord. Spatial mapping of trunk muscle activity in response to thoracic ESS may guide future targeted spinal stimulation applications, both invasively and non-invasively, for restoring trunk stability and control following neuromuscular impairment

## 2. Methods

### 2.1. Participants

Experimental data were collected in thirteen individuals with chronic pain conditions and no diagnosed neurological disorders aside from pain-related dysfunction. Each participant was undergoing clinically indicated surgery for the implantation of an epidural spinal stimulator as part of their chronic pain syndrome treatment. All participants provided informed written consent to the experimental procedures. These procedures were performed in accordance with standard of care protocols in the operating room and were conducted under research protocol Pro00023336, approved by the Institutional Ethics Review Board of the Houston Methodist Research Institute. All research was conducted in accordance with the principles embodied in the declaration of Helsinki and in accordance with local statutory requirements. Data from two participants were excluded from the analysis due to inconsistencies in stimulation frequency or amplitude range. As such, data from eleven participants (10 female and 1 male; age: 59 ± 11 years; weight: 75.1 ± 11.7 kg; height: 163 ± 10 cm) were analyzed. A summary of the participant characteristics is presented in supplementary material S1.

### 2.2. Delivery of ESS

A Spectra wavewriter spinal cord stimulator (Boston scientific, Marlborough, MA, USA) or Senza Trial Stimulator TSM1000 (Nevro, Redwood City, CA, USA) were used to generate the electrical stimulation pulses. ESS was delivered via implanted, current-controlled, 16-channel (Artisan MRI Surgical Lead, SC-8216-50, Boston scientific, Marlborough, MA, USA, or Nevro Surpass Surgical Lead, LEAD3005-50B, Nevro, Redwood City, CA, USA) or 32-channel (CoverEdge 32 Surgical Lead, SC-8336-50, Boston Scientific, Marlborough, MA, USA) electrode arrays. Electrode array specifications are listed below in table 1.

All electrodes were placed on the dorsal dura approximately on the midline within the T4 to T10 vertebrae level range, employing a laminectomy approach. Hereafter, individual vertebral levels between T4 through T10 will be referred to by their respective letter and number (e.g. ‘T5’ will denote the T5 vertebral level). The stimulator and electrode array type were tailored to patient preferences, whereas the placement and stimulation pulse width were aligned with the surgeon’s specifications for chronic pain treatment, as summarized in table 1 and supplementary material S1. X-ray images of the electrode array placement for each participant are also provided in supplementary material S1.

**Figure 1.**
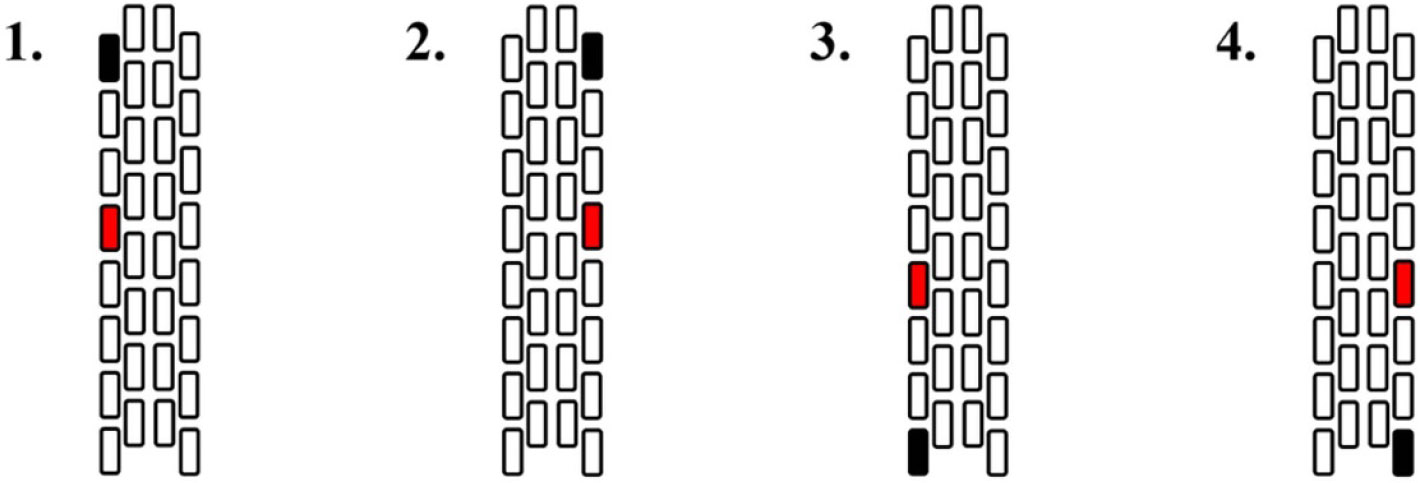
Electrode configurations: (1) left rostral, (2) right rostral, (3) left caudal, and (4) right caudal. The cathode is depicted in black, and the anode is depicted in red. The cathode was assumed to be the stimulation location as spinal structures near the cathode are most targeted by ESS [32-34]. The image shows a 32-channel array; however, the same configurations were tested for a 16-channel array using the left and right columns.

Each participant was placed in prone position, supported at the head, shoulders, and hips. Monophasic ESS was applied at 2 Hz while two parameters were systematically explored: (1) anode and cathode location; and (2) stimulation amplitude. The electrode configurations shown in figure 1 were used, in randomized order, while stimulation amplitude was continuously increased in 1.0 mA increments from 1.0 mA up to 10.0 mA (or until the ESS-evoked response plateaued). Between 3 and 65 stimuli were delivered for each stimulation amplitude. Once all trials for the maximum stimulation amplitude were completed, the next electrode configuration was tested after a no-stimulation period of 20 s or more. In total, stimulation was delivered for approximately five minutes, after which the experimental session was completed. For each participant, the pulse width was either 300 or 350 *μ*s, according to the requirements for treatment of chronic pain, as listed in supplementary material S1. To account for the variation in pulse width, we used applied charge [*μ*C], the product of stimulation amplitude and pulse width [31], as the independent variable in subsequent analyses. Furthermore, since ESS mostly targets spinal structures near the cathode [32-34], its location was considered the stimulation location.

### 2.3. Experimental data acquisition

Trigno Avanti wireless surface EMG electrodes (Delsys Inc., Natick, Massachusetts, USA) were placed on muscles relevant to trunk stability and control. EMG electrodes were placed bilaterally on the EO [16, 35-37], IO [35, 36], and RA [16, 35-37] muscles. Two surface EMG electrodes were placed unilaterally on the body side opposite to the operating surgeon on the erector spinae muscles at the levels corresponding to the sixth and seventh thoracic vertebrae (EST7) [16], and the second and third lumbar vertebrae (ESL3) [16, 36, 37]. This corresponded to placement on the left side for 8 participants and the right side for 3 participants. Note that the specific locations of the studied muscles can be found in classical textbooks such as in *Anatomy & Physiology* [38]. Two pairs of subdermal needle EMG electrodes of 20 mm length (Rhythmlink Columbia, SC, USA) were placed by an intraoperative electrophysiologist on the midline between T2 and T3 (T2/T3), and on the midline between T5 and T6 (T5/T6), with an interelectrode distance of 4 cm. These needle electrodes were used for ESS stimulus artifact identification. Details on electrode placement are presented in supplementary material S2. Data from the EMG electrodes were sampled at 2 kHz using a 16-channel PowerLab data acquisition system (Model 16/35, AD Instruments, Sydney, NSW, Australia).

### 2.4. Experimental data processing and analysis

#### Data Extraction and Processing:

Using LabChart (version 8.1.19, AD Instruments, Otago, New Zealand), EMG time series data from three trials (i.e., the minimum across participants) were exported for each electrode configuration and stimulation amplitude. The first three consecutive trials at the corresponding stimulation configuration and stimulation amplitude were selected. We ensured that, for the selected trials, the stimulation artifact was unobstructed in the recordings of at least one of the two needle electrodes. If the stimulation artifact was obstructed in a selected trial, the next consecutive trial was selected. The time series data from all participants were demeaned using the average of the baseline activity calculated from the first 100 ms of exported data, starting approximately 300 ms prior to the stimulus. All data analysis was performed in MATLAB (ver 2021b, MathWorks, Natick, MA, USA) using a custom-made algorithm.

#### Characterization of ESS-Evoked Motor Responses:

ESS-evoked responses were characterized by the metrics peak-to-peak amplitude, motor threshold (MT), and latency using the collected EMG data. All metrics were averaged across three trials. Muscles and representative samples of associated EMG data excluded from further analysis (e.g. due to movement artifacts) are presented in supplementary material S2. Peak-to-peak amplitude of the ESS-evoked responses was calculated as the maximum minus minimum response voltage within a 50 ms post-stimulus window without considering the stimulus artifact. Recruitment curves of muscle responses were created for each participant, identifying the relationship between applied charge and average peak-to-peak EMG response amplitude. MT intensity was defined as the smallest applied charge that resulted in an ESS-evoked response with a peak-to-peak amplitude greater than 20 *μ*V in all three trials [19]. Muscle responses that did not reach 20 *μ*V within the tested range of applied charge were deemed ‘non-responsive’. Latency of ESS-evoked responses was identified using thresholding relative to a baseline band (baseline ± three times the standard deviation [24]). Non-responsive muscles were omitted from latency analysis. Activation of the EST7 muscle occurred at approximately the same time as the stimulation artifact; therefore, latency values could not be calculated for this muscle.

Based on previous studies, ESS-evoked responses may be divided into early latency response (ER) and medium latency response (MR) components. ER and MR components correspond to the activation of efferent and afferent pathways, respectively [7, 24, 39]. At lower stimulation intensities, ESS is believed to produce an MR component through activation of afferent pathways in the spinal cord. The latency of the MR will remain constant with increasing stimulation intensity. At higher stimulation intensities, a response with a shorter latency may occur, which suggests additional activation of efferent pathways in the spinal cord and, therefore, an ER component [7]. If ER responses were detected in the data, an analysis of growth in ER and MR was performed. For a given muscle, the start of the ER window was defined as the response onset at the highest applied charge for a given electrode configuration [24]. The end of the ER window and the start of the MR window were defined as the response onset at the lowest applied charge that induced a response for a given electrode configuration. Once the ER and MR intervals were defined, the peak-to-peak amplitude of the response within each interval was calculated [24].

## 3. Results

### 3.1. Effect of stimulation location on muscle activity amplitude and selectivity

**Figure 2.**
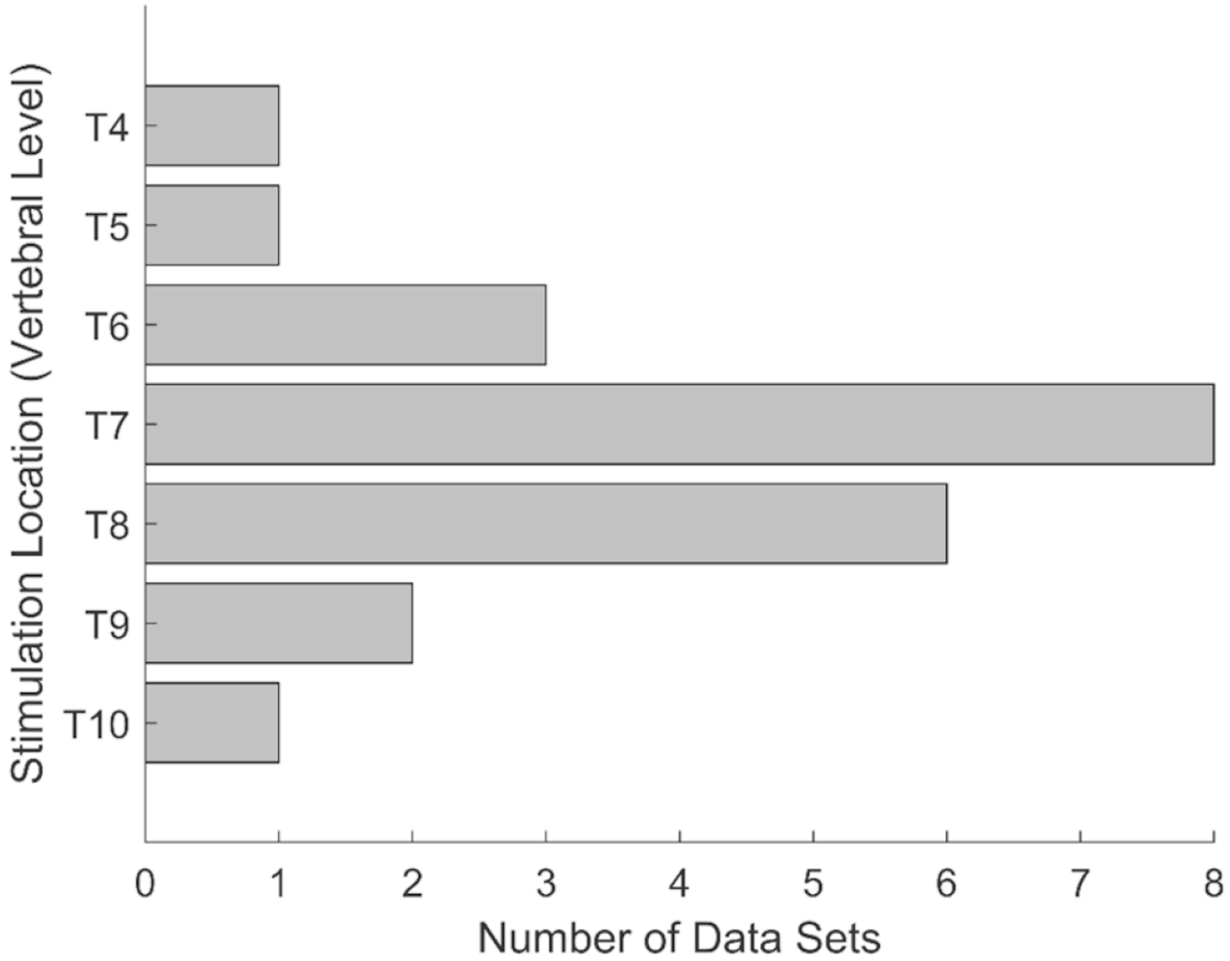
Distribution of the tested stimulation locations. Stimulation locations ranged from T4 to T10, with the greatest number of data sets occurring for T7 and T8.

Data from two participants were excluded from the analysis due to inconsistencies in stimulation parameters: one received ESS at 2 mA and 3 mA only (for these, no muscle responses were recorded due to the low stimulation amplitude), and the other received ESS at 40 Hz instead of 2 Hz. Figure 2 presents the distribution of stimulation locations tested across the eleven participants. For each participant, the electrode array was placed adjacent to two vertebral levels within the T4 to T10 range, providing two stimulation locations along the rostrocaudal axis.

**Figure 3.**
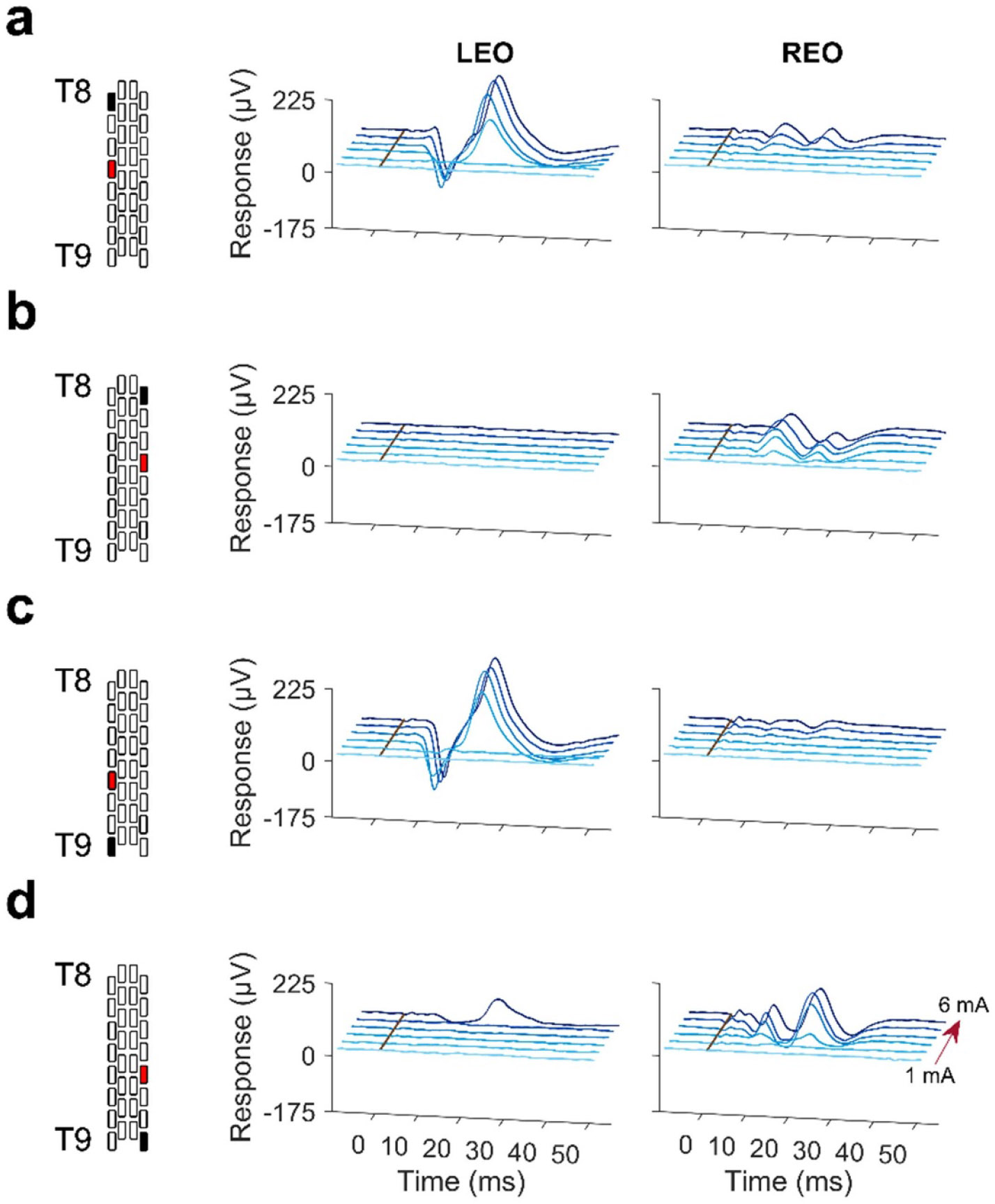
Representative ESS-evoked responses from one participant (P9) for the left and right external obliques (LEO and REO), with the electrode array placed above T8 and T9. The stimulating cathode is represented in black, and the anode in red. Waveforms, averaged across three trials, are depicted for increasing stimulation amplitudes (1–6 mA in 1 mA increments, represented by the red arrow) and when delivering stimulation above: (a) left T8, (b) right T8, (c) left T9, and (d) right T9. Stimulation occurs at zero milliseconds, represented by the brown line. Representative responses for the same participant from all other recorded trunk muscles can be found in supplementary material S3.

In figure 3, representative muscle responses from one participant induced by ESS at varying stimulation location and amplitude are presented for the left and right EO (LEO and REO). It can be seen that the manipulation of stimulation location and amplitude produced observable differences in the waveforms of the ESS-evoked responses. Overall, the evoked potentials were distinguishable from the stimulation artifact for the abdominal (i.e., EO, IO, and RA) and ESL3 muscles, whereas the stimulation artifact often occluded the evoked potentials for the EST7 muscle (see supplementary material S3, figure S3-3). Based on a visual inspection, response latency appeared consistent across trials and stimulation amplitudes for all abdominal muscles. The same trend was seen in all other participants. Note that representative ESS-evoked responses from the muscles not presented in figure 3 can be found in supplementary material S3.

**Figure 4.**
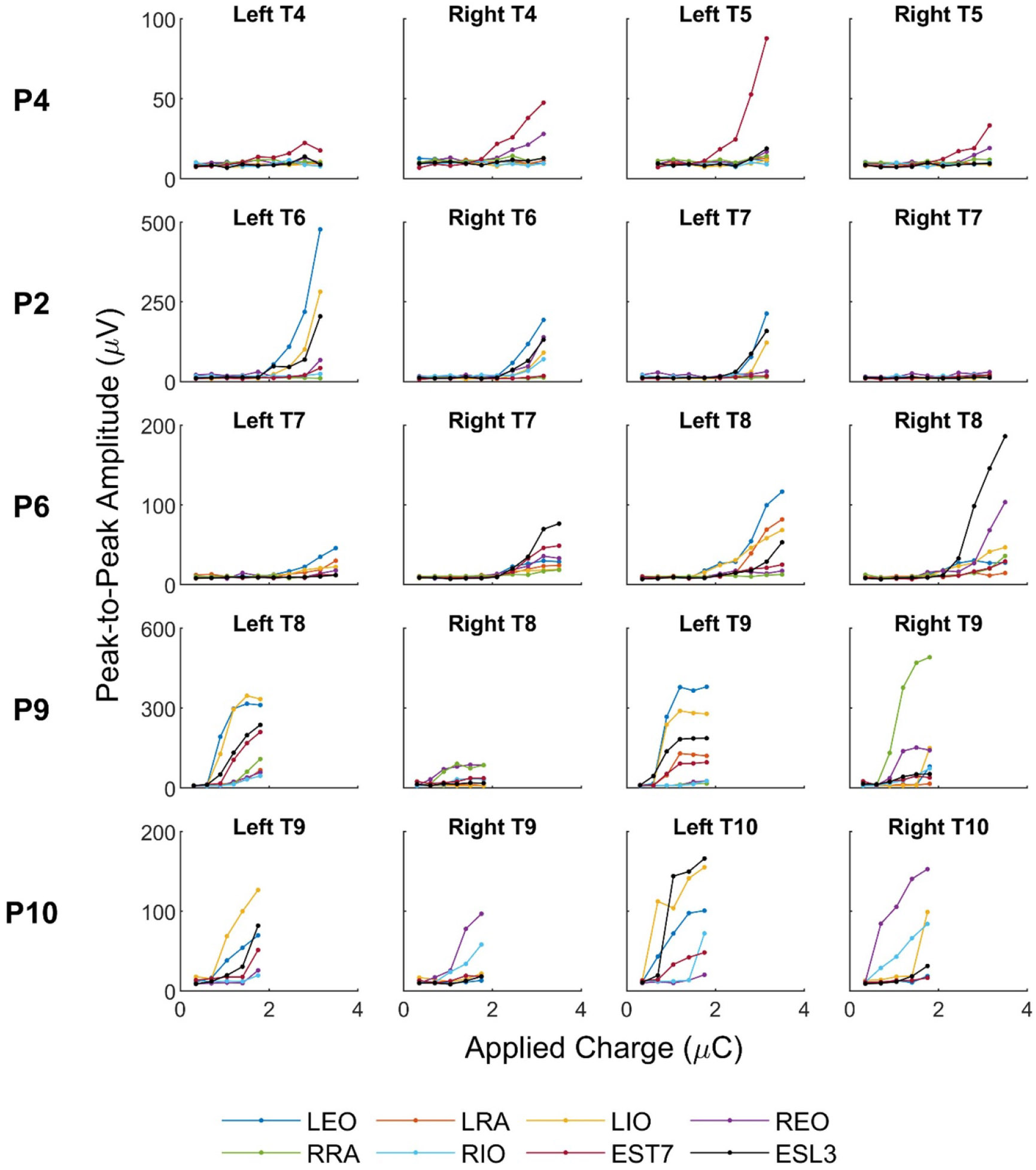
Representative recruitment curves of muscle responses during stimulation above T4 and T5 (P4), T6 and T7 (P2), T7 and T8 (P6), T8 and T9 (P9), and T9 and T10 (P10). Peak-to-peak amplitudes of the left and right external obliques (LEO and REO), internal obliques (LIO and RIO), rectus abdominis (LRA and RRA), and erector spinae at T7 and L3 (EST7 and ESL3) are shown in dependence of applied charge. Peak-to-peak amplitude values were averaged across three trials. Recruitment curves of muscle responses from the remaining six participants can be found in supplementary material S4.

Figure 4 presents the recruitment curves of muscle responses, demonstrating the relationship between applied charge and peak-to-peak amplitude of the evoked muscle responses during ESS delivered at different locations over the thoracic spine. Based on a visual inspection, ESS-evoked responses in the trunk muscles may be characterized by increasing peak-to-peak amplitude beyond MT. At greater applied charge, a subset of recruitment curves plat-eaued, likely representing saturation of motor unit recruitment. Within the range of applied charge tested, the responses recorded in the abdominal muscles (EO, IO, and RA) and the ESL3 muscle increased the most toward a plateau when stimulating caudal to T7. The responses recorded in the EST7 muscle increased at all stimulation levels tested. Individual MT values varied depending on the muscle and stimulation location, which motivated further analysis of the MT data.

**Figure 5.**
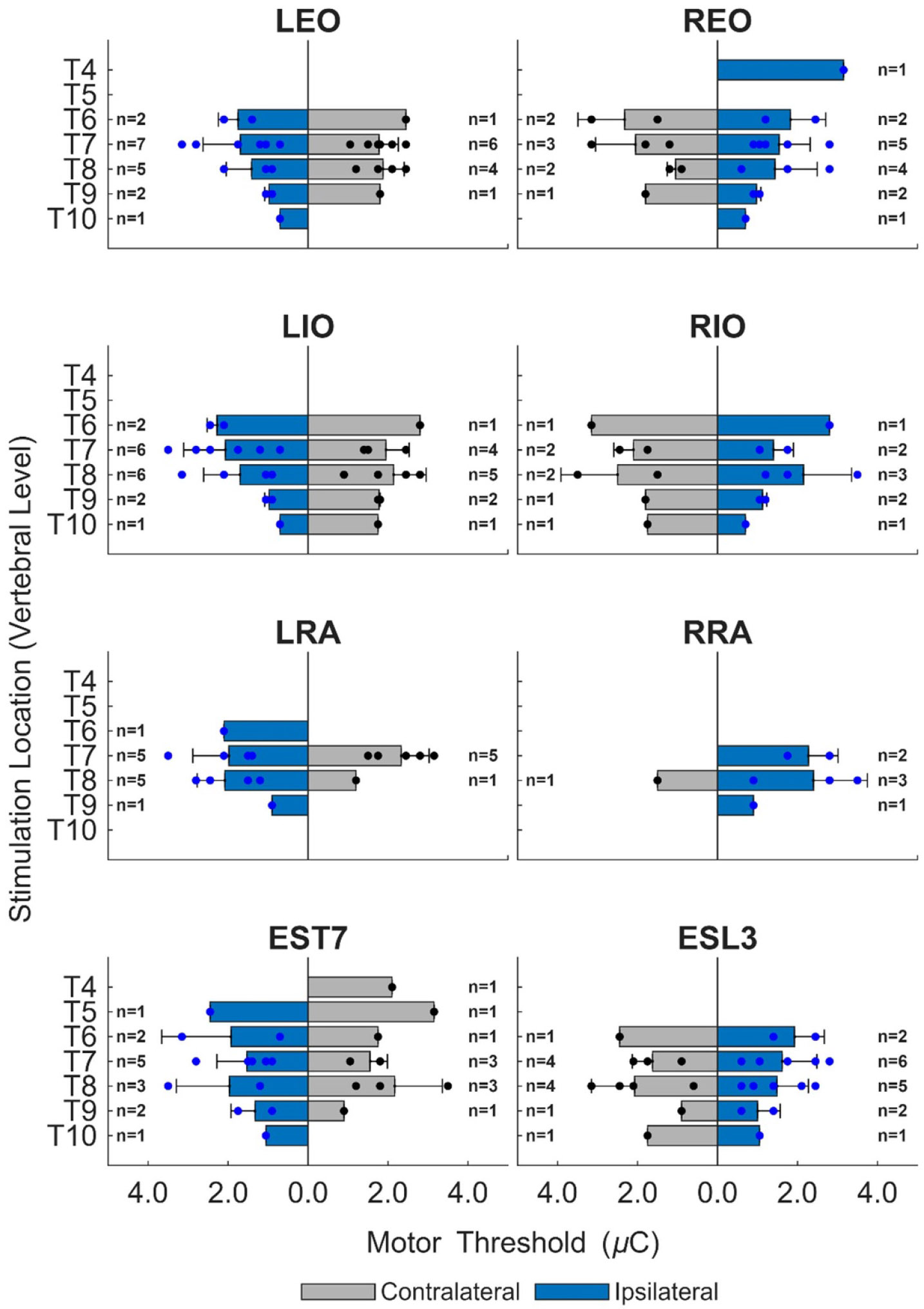
Effect of stimulation location on MT. Stimulation was delivered at different locations along the rostrocaudal axis of the spine (T4 to T10) and along the mediolateral axis of the spine (left and right side of the electrode array, represented accordingly as ipsilateral and contralateral). Shown are values at each stimulation location and across participants (mean + one standard deviation). Non-responsive muscles were omitted.

Figure 5 demonstrates the relationship between MT and stimulation location along the rostrocaudal and mediolateral axes. Muscle responses were induced by ESS caudal to T5 in the EO (*n* = 9), IO (*n* = 9), RA (*n* = 7), and ESL3 (*n* = 8) muscles. Responses in the EST7 (*n* = 9) muscle were observed for stimulation at all locations. Individual MT values varied by stimulation location and muscle, with recorded responses demonstrating lower MTs for ipsilateral stimulation (EO: 1.5 ± 0.8 (*n* = 9, 32 datapoints), IO: 1.7 ± 0.9 (*n* = 9, 26 datapoints), RA: 2.0 ± 0.9 (*n* = 7, 18 datapoints), EST7: 1.7 ± 0.9 (*n* = 9, 14 datapoints), ESL3: 1.5 ± 0.7 *μ*C (*n* = 8, 16 datapoints)) compared to contralateral stimulation (EO: 1.9 ± 0.6 (*n* = 8, 20 datapoints), IO: 2.1 ± 0.7 (*n* = 9, 20 datapoints), RA: 2.1 ± 0.8 (*n* = 7, 7 datapoints), EST7: 1.9 ± 0.9 (*n* = 6, 10 datapoints), ESL3: 1.8 ± 0.8 *μ*C (*n* = 8, 11 datapoints)). On average, a 30% lower MT was identified for ipsilateral compared to contralateral stimulation. For all recorded muscles, the lowest MT was observed during ipsilateral stimulation caudal to T8 (figure 5). For T9 and T10, contralateral stimulation did not evoke a response in the RA (T9), EO (T10), and EST7 (T10) muscles.

**Figure 6.**
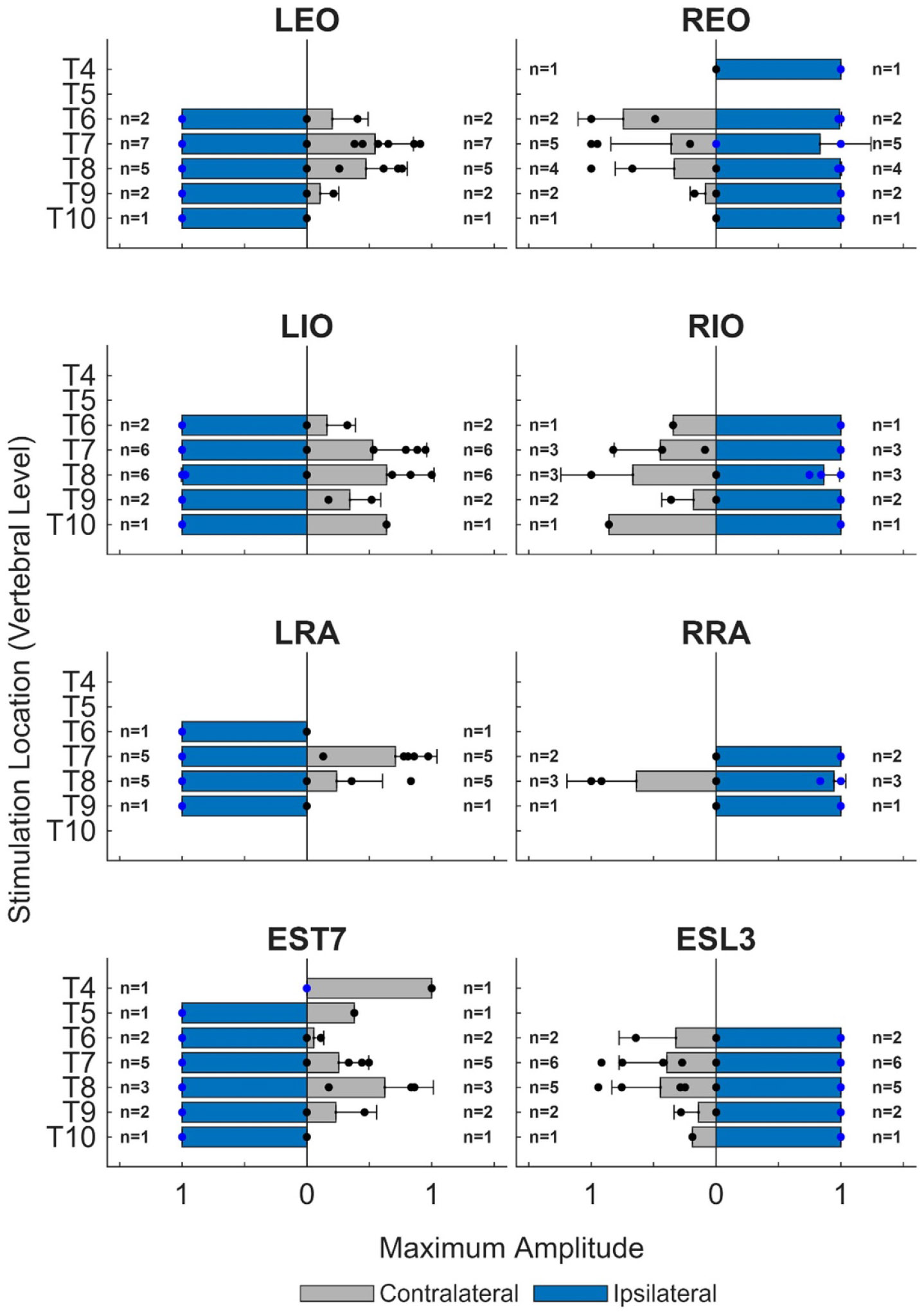
Maximum ESS-evoked response at each stimulation location in dependence of ipsilateral versus contralateral stimulation. For each stimulation location along the rostrocaudal axis of the spine, peak-to-peak amplitudes were normalized to the maximum response for each participant and muscle. Values across participants (mean + one standard deviation) at each stimulation location are shown. Non-responsive muscles were omitted.

Figure 6 presents the dependency of the maximum amplitude of the ESS-evoked responses in trunk muscles (across all stimulation amplitudes) on ipsilateral versus contralateral stimulation location. For each stimulation location along the rostrocaudal axis of the spine, peak-to-peak amplitudes were normalized to the maximum response for each participant and muscle. Ipsilateral stimulation induced approximately 2.4 times greater maximum response amplitudes compared to contralateral stimulation in all muscles and stimulation locations. At some stimulation locations, contralateral stimulation did not evoke a response which may, in part, be the result of the larger distance to contralateral dorsal roots and/or limitations associated with the size of the electric field produced for the tested range of applied charge. Some participants demonstrated greater responses from contralateral stimulation compared to ipsilateral stimulation. This was seen in six participants and only in certain muscles: P1 (REO), P2 (REO), P7 (LIO and REO), P8 (RIO), and P9 (RRA and RIO). This was also seen in P4 (EST7) where a response was only observed from contralateral stimulation. However, this muscle experienced a low-amplitude response, close to the 20 *μ*V threshold; therefore, normalization made it appear like a more favorable response.

### 3.2. Effect of stimulation location on muscle activity timing

**Figure 7.**
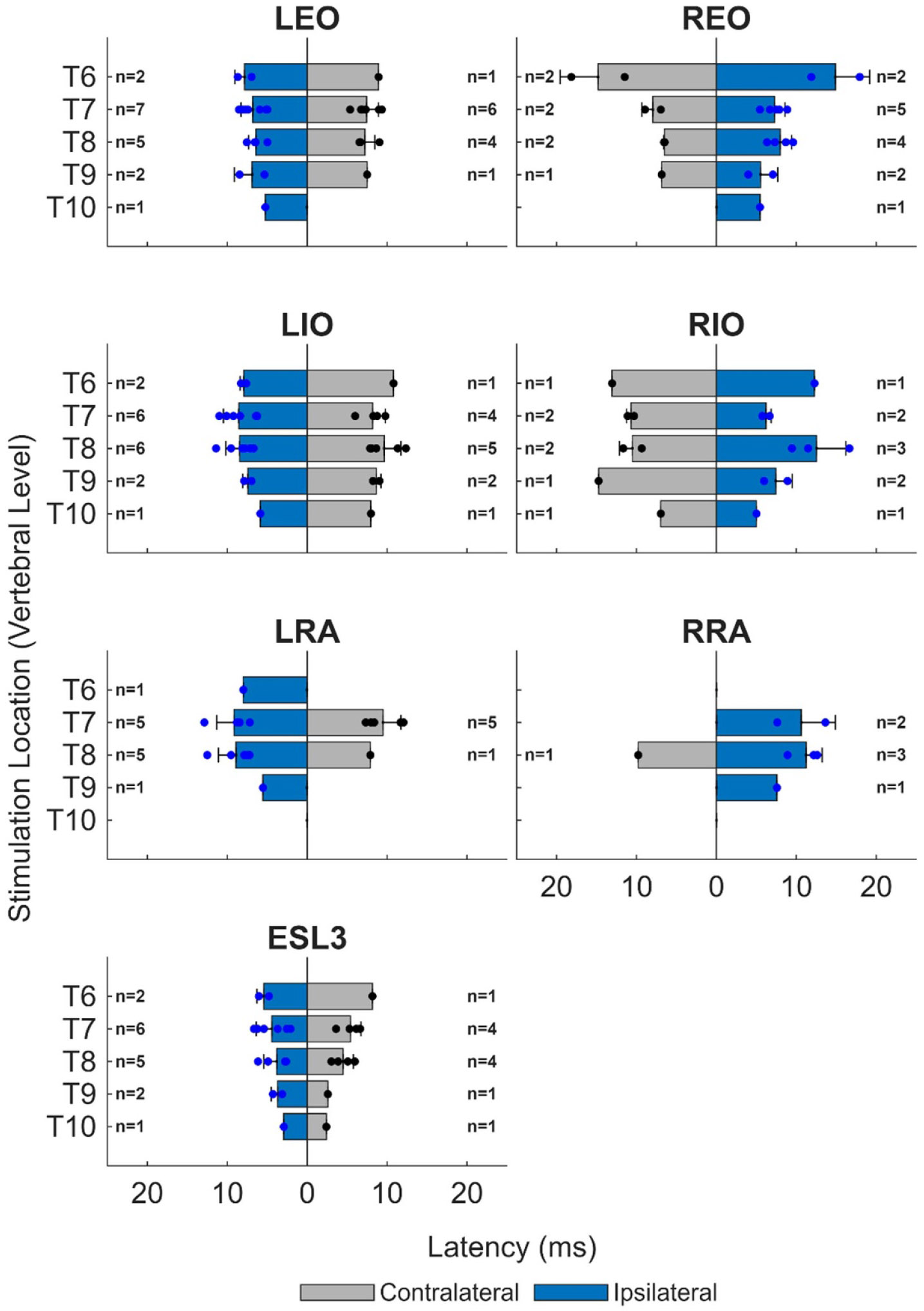
Latency of ESS-evoked responses at maximum stimulation amplitude for each stimulation location along the rostrocaudal axis of the spine (T6 to T10) and along the mediolateral axis of the spine (left and right side of the electrode array, represented accordingly as ipsilateral and contralateral). Latency values at each stimulation location were averaged across participants (mean + one standard deviation). Non-responsive muscles were omitted.

Figure 7 presents, for the maximum stimulation amplitude, the relationship between the latency of ESS-evoked responses in trunk muscles and stimulation location along the mediolateral and rostrocaudal axes. The latency values varied along the rostrocaudal axis, and exhibited a general trend toward shorter response latencies from ipsilateral (EO: 7.4 ± 2.6, IO: 8.5 ± 2.6, RA: 9.2 ± 2.4, ESL3: 4.2 ± 1.6 ms) compared to contralateral stimulation (EO: 8.2 ± 2.8, IO: 9.5 ± 2.1, RA: 11.2 ± 4.1, ESL3: 4.8 ± 1.8 ms). We identified an average difference of 0.9 ms between ipsilateral and contralateral stimulation (EO: 0.6, IO: 1.2, RA: 1.1, ESL3: 0.6 ms). Visually, there was a trend toward shorter response latencies from stimulation more caudal on the spine. Responses recorded in the ESL3 muscle had shorter latencies at maximum stimulation compared to the abdominal muscles (figure 7).

**Figure 8.**
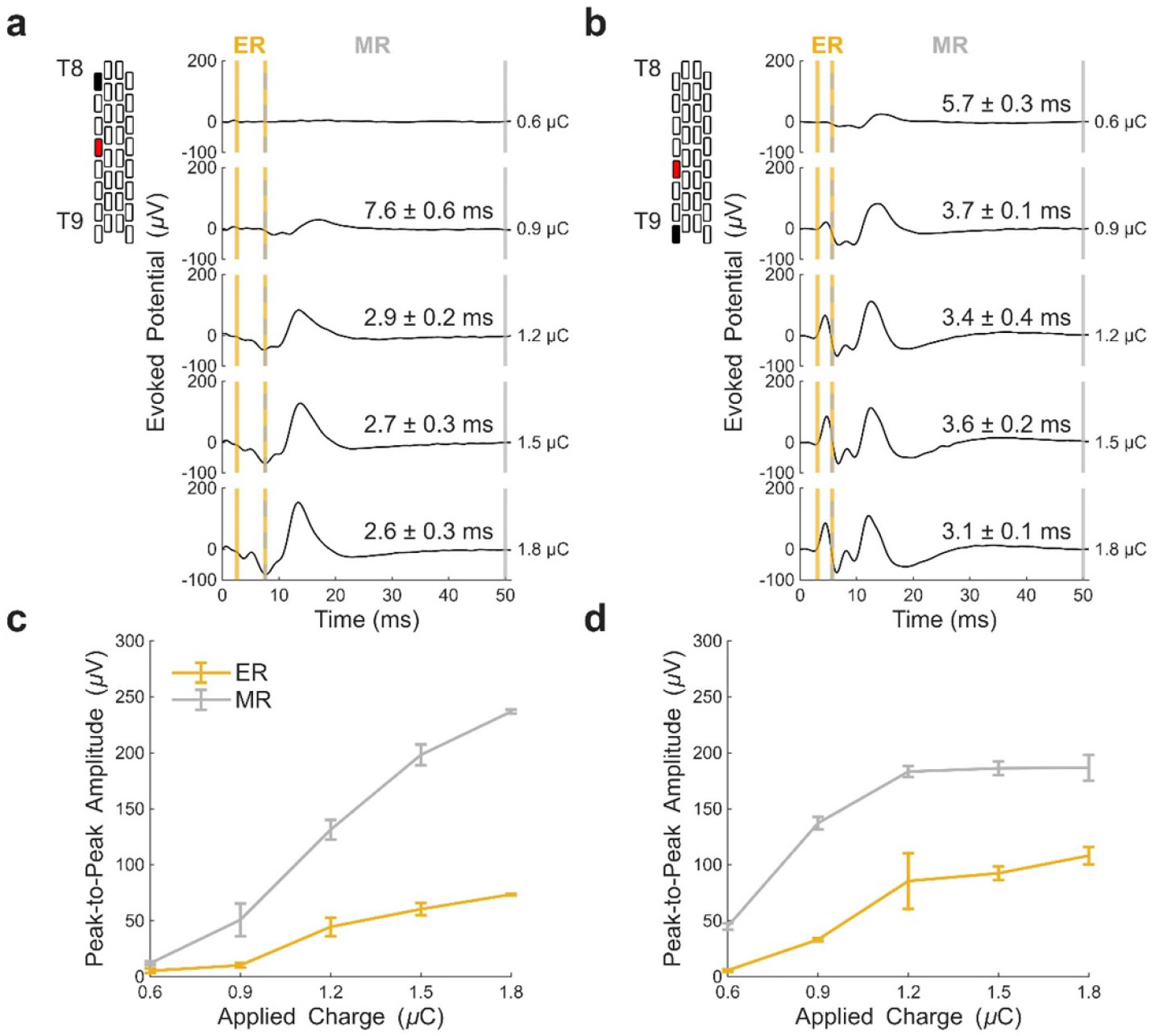
Latency analysis for early responses (ER) and medium responses (MR) recorded in the ESL3 muscle for P9. ESS-evoked responses with increasing applied charge are shown for ipsilateral stimulation above: (a) left T8, and (b) left T9. Latency values averaged across three trials (mean ± one standard deviation stated on each waveform) decreased at higher applied charge. The start of the early response window (vertical solid yellow line) is identified as the latency at the highest applied charge for a given electrode configuration. The start of the medium response window (vertical dashed yellow and grey line) is identified as the latency at the lowest applied charge that induced a response for a given electrode configuration. The end of the MR window (vertical solid grey line) is 50 ms. In (c) and (d), the peak-to-peak amplitudes of the ER and MR components are compared when increasing applied charge. All waveforms and obtained metrics were averaged across three trials. The latency analysis for P10 can be found in supplementary material S5.

In figure 8, the potential presence of ER and MR responses in the ESL3 muscle is presented for P9. An MR was detected at lower stimulation amplitudes. At greater stimulation amplitudes, a decrease in response latency was observed, which suggests the presence of an ER component. ER and MR responses were also seen in the ESL3 muscle for P10 (supplementary material S5).

## 4. Discussion

The purpose of this study was to investigate the effect of ESS delivered to the thoracic spinal cord on the activity of trunk muscles involved in postural control and stability. The results demonstrate that the amplitude, MT, and timing of ESS-evoked responses in trunk muscles depend on the location of ESS. Furthermore, the results suggest activation of both afferent and efferent spinal pathways when applying thoracic ESS.

### 4.1. Side-selectivity of trunk muscle activity enabled by thoracic ESS

Lateral ESS induced ipsilateral and contralateral muscle responses; however, lower MTs, greater response amplitudes, and shorter latencies occurred during ipsilateral stimulation. Similarly, recruitment curves of ESS-evoked muscle responses were more likely to reach a plateau from ipsilateral stimulation. It is important to note that this side-selectivity was seen in trunk muscles during stimulation at the thoracic vertebral levels ranging from T6 to T10. In addition, it is consistent with findings from previous studies investigating the location-specific effects of lumbar ESS that demonstrated greater muscle response amplitudes in ipsilateral trunk and lower limb muscles [27, 40, 41]. Stimulation caudal to T8 did not produce a response in the contralateral RA muscle, and stimulation caudal to T9 did not produce a response in the contralateral EO and EST7 muscles. However, at these two levels, stimulation was only increased to 6 mA (P9; equivalent applied charge: 1.8 *μ*C) and 5 mA (P10; equivalent applied charge: 1.75 *μ*C), respectively; therefore, contralateral responses may still be achieved at higher stimulation amplitudes, as seen at other levels.

Side-selectivity is beneficial for applications that require side-specific muscle activity, such as during ESS-assisted stepping following SCI [41-43]. This need for side-specific muscle activity may also be valuable in trunk control applications, for example enabling unilateral trunk excursion during lateral reaching [27]. Using lower frequency stimulation (0.5 Hz), Rowald *et al* delivered ESS at T12 to L2 to assess side-selectivity of trunk muscles [27]. Lateral stimulation at T12 induced responses in ipsilateral upper RA, obliques, and the quadratus lumborum, which, when translated to a higher frequency stimulation, resulted in ipsilateral trunk movements [27]. For future applications, the present work suggests lateral thoracic ESS at lower stimulation intensities to be able to target ipsilateral trunk muscles while preventing contralateral muscle activation.

Irrespective of the important findings discussed above, this study is limited in its ability to assess side-selectivity in dependence of stimulation distance from the midline. Some participants presented asymmetrical ESS-evoked responses and, at times, greater responses from contralateral stimulation. This, in part, may be the result of the electrode array placement (i.e. inadvertent deviations from the midline) or variations in the placement of EMG electrodes for left and right muscles. Previous studies also reporting an asymmetry between left and right responses [19, 20] attributed this to anatomical variability, including uneven muscle innervation [44], asymmetric musculature [45], or asymmetric dorsal root ganglia position and morphology [46]. Some participants in the present study were also diagnosed with lumbar radiculopathy, which may be linked to asymmetric atrophy of paraspinal muscles [47]. Based on the geometry of the electrode array, and assuming central placement, lateral stimulation occurred approximately 2 mm from the midline. However, simulation results suggest placing the electrodes 4.7 mm from the midline to allow for the greatest side-selectivity while accounting for mediolateral variations in electrode array positioning [27].

### 4.2. Rostrocaudal manipulation modulates amplitude and selectivity of trunk muscle activity

Previous work has shown that manipulation of stimulation location along the rostrocaudal axis modulates the amplitude and selective activation of lower limb, abdominal, and trunk muscles [24, 27]. For example, upper abdominal muscles exhibited a greater response amplitude from ESS targeting T12 compared to L1/L2 [27]. In the context of trunk control and stability, the same modulation has been used to improve functional outcomes [26]. ESS targeting T12 to L1/L2 induced lateral trunk excursion, however, selective activation of trunk muscles without unintentional leg muscle activation was better achieved with stimulation in the more rostral region of the electrode array, corresponding to T12 [27]. ESS electrode placement within T11 to L1 improved forward reaching following complete, thoracic SCI, with stimulation from the caudal region of the electrode array enabling the greatest improvement [26]. Interestingly, the same electrode placement did not improve lateral reach distance [26]. These findings demonstrate that localized stimulation along the rostrocaudal axis targets specific motor pools projecting to trunk muscles, resulting in varying functional outcomes. However, these findings also highlight the need for further testing of ESS at additional vertebral levels within the thoracic spine, as addressed in the present work.

The results from this study demonstrated that ESS along the rostrocaudal axis within the thoracic region of the spine, and particularly between T4 and T10, can activate trunk muscles. The anterior abdominal (EO, IO, and RA) and ESL3 muscles were activated from stimulation at and more caudal to T6. Stimulation rostral to T6 did not induce muscle responses in the abdominal muscles within the range of applied charge tested. This was expected as current anatomical knowledge suggests innervation of each above muscle to lie within the T7 to L1 spinal segment range, which approximately aligns with the vertebral segment range between the caudal region of T5 and the rostral region of T11 [29, 30]. The erector spinae muscle recorded at T7 was activated at all stimulation locations tested. In addition, multiple muscles were activated during ESS delivered at multiple locations between T6 and T10, demonstrating the ability to target an ensemble of trunk muscles with one stimulation location.

Despite differences in expected spinal innervation regions, all trunk muscle responses demonstrated a general trend towards a lower MT and shorter response latency from stimulation in the lower thoracic region compared to the midthoracic region. This trend was even seen in the EST7 muscle; however, results may be impacted by response occlusion from stimulation artifacts more rostral on the spine. In this context, it is important to note that activation thresholds of dorsal roots are affected by the location, orientation, and curvature of the dorsal root with respect to the electric field [48-51]. Lower thresholds resulting from lower thoracic stimulation may also result from the spread of the electric field reaching a greater number of dorsal roots projecting to trunk muscles. This idea is feasible considering stimulation projecting laterally likely targets nearby ascending dorsal roots, in addition to the dorsal root entry zone at the level near the cathode [52]. Contrary to the results for MT and latency, there was no clear trend when investigating the effect of rostrocaudal manipulation on maximum response amplitude. In fact, comparing EMG amplitudes across individuals has limited value; therefore, amplitudes of evoked motor responses can only be compared between rostral and caudal stimulation *within* the electrode array (i.e. across different vertebral levels) for each participant. Due to the close proximity between the rostral and caudal electrode within the electrode array, the electric field from rostral stimulation may have excited dorsal roots neighboring the caudal electrode, and vice versa, reducing the impact of rostrocaudal manipulation.

Our findings may enable objectively guided electrode placement in future applications, which require targeting of specific motor pools. However, methodological considerations may limit accurate comparisons of stimulation location along the rostrocaudal axis; hence, generalizations must be viewed with caution. Anatomic variability in the alignment of the spinal segments and vertebral levels [53] as well as varied placement of electrode arrays, even across participants tested at the same stimulation location, impact the accuracy of rostrocaudal comparisons. Minute variation in rostrocaudal array placement across participants can impact the location of the cathode relative to the dorsal root entry zone, which has been shown to influence the amplitude of an evoked muscle response even more than manipulation of vertebral level [54]. Signal transmission speed and distance are variable across participants, e.g. due to differences in height and age, limiting latency comparisons [55, 56]. MT as a metric for inter-participant comparisons is limited due to variability in dorsal root anatomy (i.e. fiber diameter, innervation angle), which affects excitability [48, 49], and/or variability in tissue layer thickness, which affects the measured amplitude through surface EMG [57]. Lastly, our results are based on a different number of data sets at each stimulation location, which limits the generalizability of our results.

### 4.3. Trunk muscle activation pathways from thoracic ESS

In the present work, preferential activation of ipsilateral trunk muscles supports the idea that lateral ESS off the midline mainly targets dorsal roots as they ascend prior to or at the junction with the spinal cord. Contralateral responses at higher intensities and longer latencies suggest additional activation of nearby inter-neuronal structures, dorsal columns, or contralateral dorsal roots due to the spread of the electrical field [19]. The present work showed a difference between ipsilateral and contralateral latencies of approximately 1 ms, varying by muscle and stimulation location. This agrees with previous findings reporting an approximately 2 ms difference, which could be explained by contralateral activation through crossed reflex pathways in the studied abdominal muscles [58, 59].

Spinal stimulation studies differentiate between ER and MR responses [7, 24, 39, 54]. ER and MR responses have been induced in lower limb muscles resulting from lumbosacral ESS, with ER responses only appearing at higher stimulation intensities [24, 60, 61]. ER responses, at higher stimulation intensities, are attributed to direct motor axon activation [24, 60-62], whereas MR responses are believed to result from activation via transsynaptic pathways [7, 24, 39, 60-62]. These components are often overlapping and produce a combined waveform [24, 62]. In the present work, for stimulation at and caudal to T8, the ESL3 muscle experienced shorter latencies and overlapping waveforms at higher stimulation amplitudes. This was not seen in the abdominal muscles or EST7 muscle, however, the EST7 response was often occluded by the stimulation artifact preventing further analysis. Due to the proximity of the ESL3 muscle to the electrode array, one would expect motor axons projecting to this muscle to be closer to the electrode array when compared to abdominal muscles. These findings suggest that thoracic ESS can target both afferent and efferent pathways projecting to back muscles. However, the presumed activation of efferent pathways was only seen in two participants (P9 and P10), therefore generalizations about the response of this muscle at this stimulation location should be viewed with caution.

### 4.4. Limitations and future directions

In addition to the anatomic variability mentioned previously, the present work is limited by the pre-dominantly female and relatively small sample size which may limit the generalizability of results. Future efforts require a larger pool of data, especially above the most rostral (T4 to T5) and most caudal (T9 to T10) vertebral levels. Due to experimental time constraints, stimulation amplitude was increased from 1.0 mA to 10.0 mA in 1.0 mA increments. Future work should consider a smaller step size to enable more accurate mediolateral and rostrocaudal comparisons of muscle response amplitudes and MTs. Differences in the size and type of implanted electrode array also pose limitations; however, the interelectrode distances remained relatively constant, allowing for inter-participant comparisons.

The findings from this study demonstrate the relationship between thoracic ESS location and trunk muscle activation. This relationship may guide assistive or therapeutic applications for improving trunk muscle activation following neuromuscular impairment, such as SCI. The findings presented here must account for potential inter-individual variability in the anatomical relationship between the spinal cord and vertebral column [63, 64]. Therefore, the vertebral levels identified in this study should be regarded as approximate guides. Previous studies have demonstrated similar spinal maps for able-bodied individuals and individuals with upper motoneuron disorders, including SCI [52], supporting the transferability of the present results to individuals with SCI. However, since the present results were obtained in individuals with chronic pain syndrome, their interpretation for individuals with SCI should be approached with caution [47]. Moreover, individuals with SCI can exhibit diverse injury levels and varying degrees of motor neuron damage at and below the lesion site, which may contribute to variability in spinal stimulation effects on muscle activation and functional outcomes. Understanding these differences is crucial for optimizing stimulation parameters and tailoring neuromodulation strategies to maximize therapeutic benefits for individuals with SCI. It should also be noted that previous studies have demonstrated similar neural activation pathways and mechanisms for TSS and ESS [8, 65], suggesting that the results from this study may also be applicable to clinical TSS applications. Future work should determine whether TSS yields comparable outcomes in activating trunk muscles and further refine stimulation protocols to enhance therapeutic efficacy.

## 5. Conclusion

Motoneurons within the thoracic spinal cord were modulated through rostrocaudal and mediolateral adjustment of ESS, enabling targeted activation of trunk muscles. ESS applied between the T6 and T10 vertebrae effectively engaged trunk musculature via afferent and efferent spinal networks, with larger response amplitudes observed from ipsilateral stimulation. Ipsilateral ESS between T8 and T10 vertebrae activated trunk muscles at lower intensities and shorter latencies. These findings highlight the critical role of stimulation location on motor pool activation, emphasizing the importance of spatial mapping for optimizing spinal stimulation strategies. Ultimately, this study advances our understanding of thoracic spinal networks and provides valuable insights for refining electrode placement in future therapeutic or assistive spinal stimulation applications aimed at enhancing trunk stability and postural control following SCI.

## Supplementary Material

Supplementary

Supplementary material for this article is available online

## Figures and Tables

**Table 1.** Electrode array specifications.

Electrode type	Model	Lead shape	Number of electrode contacts	Array length (rostro-caudal)	Array width (mediolateral)	Electrode size (width × length)	Electrode spacing (longitudinal)	Electrode spacing (latitudinal)
CoverEdge 32 Surgical lead^a^	SC-8336-50	4 × 8 array	32	50 mm	9 mm	1 mm × 3.4 mm	1 mm	*Information not provided in data sheets*
Artisan MRI Surgical Lead^a^	SC-8216-50	2 × 8 array	16	45 mm	8 mm	2 mm × 3 mm	1 mm	*Information not provided in data sheets*
Nevro Surpass Surgical Lead^b^	LEAD3005–50B	2 × 8 array	16	64 mm	10 mm	1.25 mm × 3.0 mm	4.25 mm	1.0 mm

a Boston Scientific, Marlborough, MA, USA.

b Nevro, Redwood City, CA, USA.

## Data Availability

All data that support the findings of this study are included within the article (and any supplementary files).
