# Supplementary material for: Differential modulation of trunk muscle activation using thoracic epidural spinal stimulation
Source: J Neural Eng. Author manuscript; Available in PMC 2026 May 27. (PMC13215678; doi:10.1088/1741-2552/adf9a0)
Supplement: Supplementary [file NIHMS2173811-supplement-Supplementary.docx]

**Differential modulation of trunk muscle activation using thoracic epidural spinal stimulation**

**--- Supplementary Material ---**

Authors:

Sydney Schadan^1^, Alexander G. Steele^2^, Amir H. Faraji^3^, Albert H. Vette^1,4,5,*,†^, Dimitry G. Sayenko^2,†^

^1^ Department of Mechanical Engineering, University of Alberta, Donadeo Innovation Centre for Engineering, 9211 116 Street NW, Edmonton, Alberta T6G 1H9, Canada

^2^ Department of Neurosurgery, Center for Neuroregeneration, Houston Methodist Research Institute, 6550 Fannin Streat, Houston, Texas 77030, United States of America

^3^ Department of Neurological Surgery, Houston Methodist Hospital, 6565 Fannin Street, Houston, Texas 77030, United States of America

^4^ Glenrose Rehabilitation Hospital, Alberta Health Services,10230 111 Avenue NW, Edmonton, Alberta T5G 0B7, Canada

^5^ Department of Biomedical Engineering, University of Alberta, Donadeo Innovation Centre for Engineering, 9211 116 Street NW, Edmonton, Alberta T6G 1H9, Canada

* corresponding author (vette@ualberta.ca)

^†^ shared senior authorship

# **Supplementary Material S1: Participant Summary**

**Table S1-1.** Summary of participant characteristics.

| **Participant ID** | **Age**  **(years)** | **Sex** | **Height**  **(cm)** | **Weight**  **(kg)** | **Reason for Implant** |
| --- | --- | --- | --- | --- | --- |
| P9 | 78 | F | 160 | 95.0 | - Chronic pain syndrome; failed back syndrome - Persistent lumbar radiculopathy, radiculitis - Intervertebral disc disorder with radiculopathy, lumbosacral |
| P10 | 69 | M | 185 | 73.9 | - Chronic pain syndrome; post laminectomy syndrome - Intervertebral disc disorder with radiculopathy, lumbosacral |
| P11 | 68 | F | 168 | 90.7 | - Chronic pain syndrome; failed back syndrome - Intervertebral disc disorder with radiculopathy, lumbosacral |
| P12 | 60 | F | 152 | 62.5 | - Chronic pain syndrome; post laminectomy syndrome - Intervertebral disc disorder with radiculopathy, lumbosacral - Malfunction and migration of spinal cord stimulator |
| P13 | 68 | F | 172 | 79.7 | - Failed back syndrome - Intervertebral disc disorder with radiculopathy, lumbosacral |
| P14 | 44 | F | 172 | 82.1 | - Chronic pain syndrome; failed back syndrome - Intervertebral disc disorder with radiculopathy, lumbosacral |
| P15 | 66 | F | 157 | 69.9 | - Chronic pain syndrome; failed back syndrome - Persistent lumbar radiculopathy, radiculitis |
| P16 | 51 | F | 155 | 64.2 | - Chronic pain syndrome; failed back syndrome - Persistent lumbar radiculopathy, radiculitis - Intervertebral disc disorder with radiculopathy, lumbosacral |
| P21 | 49 | F | 160 | 79.3 | - Chronic pain syndrome |
| P22 | 51 | F | 157 | 70.8 | - Chronic pain syndrome |
| P26 | 46 | F | 157 | 57.6 | - Revision surgery to replace stimulator to treat pain |

**Table S1-2.** Electrode array specifications.

| **Electrode Type** | **Model** | **Lead Shape** | **Number of Electrode Contacts** | **Array Length (Rostro-caudal)** | **Array Width (Mediolateral)** | **Electrode Size (Width x Length)** | **Electrode Spacing (longitudinal)** | **Electrode Spacing (latitudinal)** |
| --- | --- | --- | --- | --- | --- | --- | --- | --- |
| CoverEdge 32 Surgical Lead^1^ | SC-8336-50 | 4 x 8 array | 32 | 50 mm | 9 mm | 1 mm x 3.4 mm | 1 mm | *Information not provided in data sheets* |
| Artisan MRI Surgical Lead^1^ | SC-8216-50 | 2 x 8 array | 16 | 45 mm | 8 mm | 2 mm x 3 mm | 1 mm | *Information not provided in data sheets* |
| Nevro Surpass Surgical Lead^2^ | LEAD3005-50B | 2 x 8 array | 16 | 64 mm | 10 mm | 1.25 mm x 3.0 mm | 4.25 mm | 1.0 mm |

^1^ Boston Scientific, Marlborough, MA, USA.

^2^ Nevro, Redwood City, CA, USA.

**Table S1-3.** Participant summary of ESS electrode placement and ESS stimulation parameters.

| **Participant ID** | **External Stimulator** | **Electrode Array** | **Electrode Contacts** | **Array Placement (Vertebral Levels)** | **Pulse Width (μs)** | **Stimulation Amplitude Range (mA)** |
| --- | --- | --- | --- | --- | --- | --- |
| P9 | Spectra WaveWriter Spinal Cord Stimulator^1^ | CoverEdge 32 Surgical Lead^1^ | 32 | T6 to T7 | 300 | 4.0 to 6.0 |
| P10 | Senza Trial Stimulator TSM1000^2^ | Nevro Surpass Surgical Lead^2^ | 16 | T6 to T7 | 350 | 1.0 to 9.0 |
| P11 | Spectra WaveWriter Spinal Cord Stimulator^1^ | CoverEdge 32 Surgical Lead^1^ | 32 | T7 to T8 | 350 | 1.0 to 6.0 |
| P12 | Spectra WaveWriter Spinal Cord Stimulator^1^ | CoverEdge 32 Surgical Lead^1^ | 32 | T4 to T5 | 350 | 1.0 to 9.0 |
| P13 | Spectra WaveWriter Spinal Cord Stimulator^1^ | CoverEdge 32 Surgical Lead^1^ | 32 | T7 to T8 | 350 | 1.0 to 8.0  *Left caudal only: 1.0 to 9.0 |
| P14 | Spectra WaveWriter Spinal Cord Stimulator^1^ | CoverEdge 32 Surgical Lead^1^ | 32 | T7 to T8 | 350 | 1.0 to 10.0 |
| P15 | Spectra WaveWriter Spinal Cord Stimulator^1^ | CoverEdge 32 Surgical Lead^1^ | 32 | T7 to T8 | 300 | 1.0 to 7.0 |
| P16 | Spectra WaveWriter Spinal Cord Stimulator^1^ | Artisan MRI Surgical Lead^1^ | 16 | T7 to T8 | 350 | 1.0 to 10.0 |
| P21 | Spectra WaveWriter Spinal Cord Stimulator^1^ | CoverEdge 32 Surgical Lead^1^ | 32 | T8 to T9 | 300 | 1.0 to 6.0 |
| P22 | Spectra WaveWriter Spinal Cord Stimulator^1^ | CoverEdge 32 Surgical Lead^1^ | 32 | T9 to T10 | 350 | 1.0 to 5.0 |
| P26 | Spectra WaveWriter Spinal Cord Stimulator^1^ | CoverEdge 32 Surgical Lead^1^ | 32 | T6 to T7 | 350 | 1.0 to 7.0 |

^1^ Boston Scientific, Marlborough, MA, USA.

^2^ Nevro, Redwood City, CA, USA.


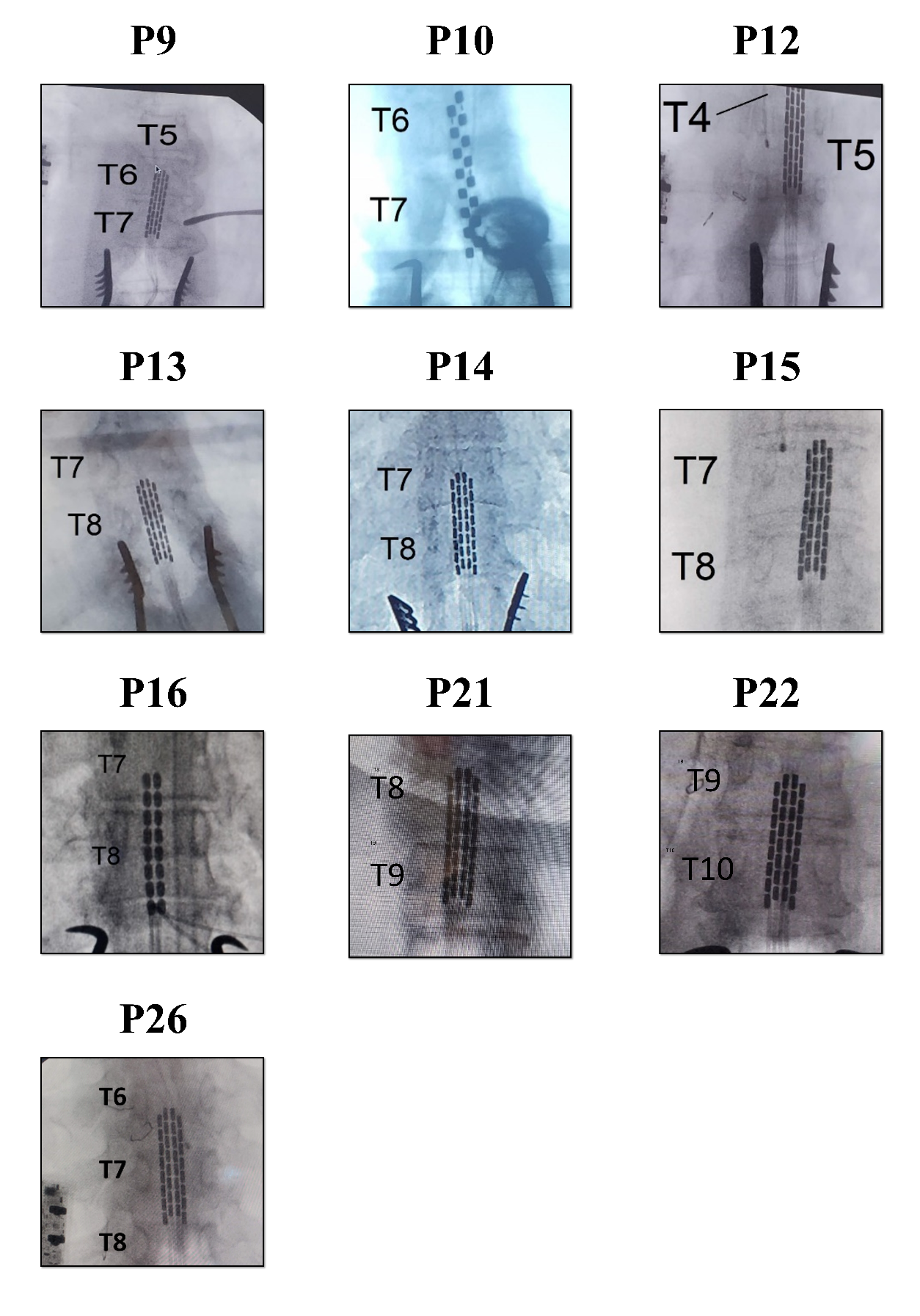


**Figure S1-1.** X-ray images of the electrode paddle placements for each participant. An x-ray image for P11’s electrode paddle placement at T7 to T8 was not available.

# **Supplementary Material S2: Muscle and EMG Exclusions**

**Table S2-1**. Guidelines for placing the EMG surface electrodes.

| **Muscle** | **Electrode Placement** | **Electrode Orientation** |
| --- | --- | --- |
| Rectus abdominis | 3 cm lateral of the umbilicus^1–3^ | Aligned vertically |
| External obliques | 15 cm lateral of the umbilicus^1–3^ | Aligned at 45 degrees off the vertical |
| Erector spinae above the T6/T7 vertebral level | 5 cm lateral of T6/T7 vertebral level^1–3^ | Aligned vertically |
| Erector spinae above the L2/L3 vertebral level | 3 cm lateral of L2/L3 vertebral level^1–3^ | Aligned vertically |
| Internal obliques | At the midpoint between the anterior superior iliac spine and the symphysis pubis, above the inguinal ligament^1,2^ | Aligned at 45 degrees off the vertical |

EMG surface electrodes were placed on trunk muscles according to the placement guidelines outlined in Table S2-1. Muscles were excluded from data analysis when the electrode was presumed to have fallen off or to have captured a movement artifact during the experimental session. An electrode that had fallen off during data collection was identified by regular oscillations in the EMG data. A motion artifact was presumed when a low-frequency response, ranging from 1 to 10 Hz^4^, was detected with onset and duration that differed from a typical response. A summary of all muscle exclusions is included in Table S2-2. A comparison of a typical RRA response to (1) when an electrode had fallen off and (2) a presumed motion artifact was present is shown below in Fig. S2-1 and Fig. S2-2, respectively.

**Table S2-2.** Muscle exclusions: RRA = right rectus abdominis; LRA = left rectus abdominis; REO = right external obliques; RIO = right internal obliques; LEO = left external obliques; EST7 = erector spinae at T6/T7 vertebra level; and ESL3 = erector spinae at L3 vertebra level.

| **Participant ID** | **Muscle(s)** | **Justification for Exclusion** |
| --- | --- | --- |
| P9 | RRA | Electrode fell off |
| P10 | LRA | Electrode fell off |
| P11 | RRA | Movement artifact |
| P13 | REO | Electrode fell off |
| P14 | RIO | Movement artifact |
| P15 | RRA, RIO | Movement artifact |
| P16 | LEO  EST7, ESL3 | Electrode fell off;  noise |
| P22 | LRA, RRA | Movement artifact |


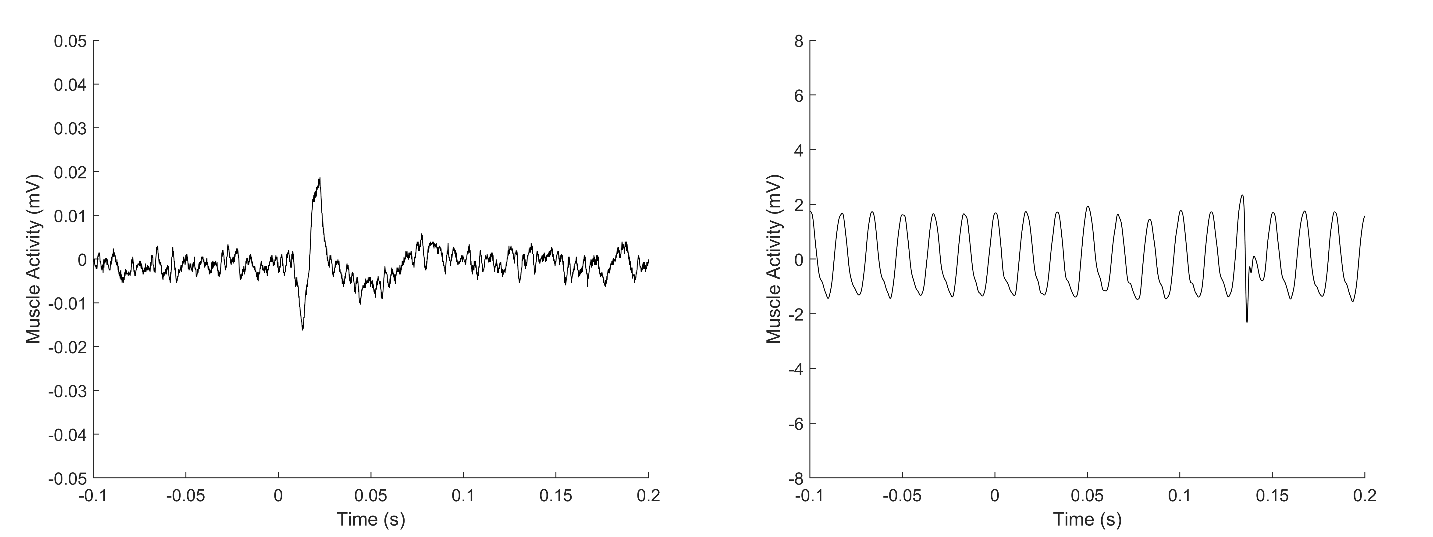


**Figure S2-1.** A typical RRA response from P14 (left) compared to the EMG signal when the electrode fell off during experimental data collection from P9 (right). The removed electrode exhibits repeated oscillations.


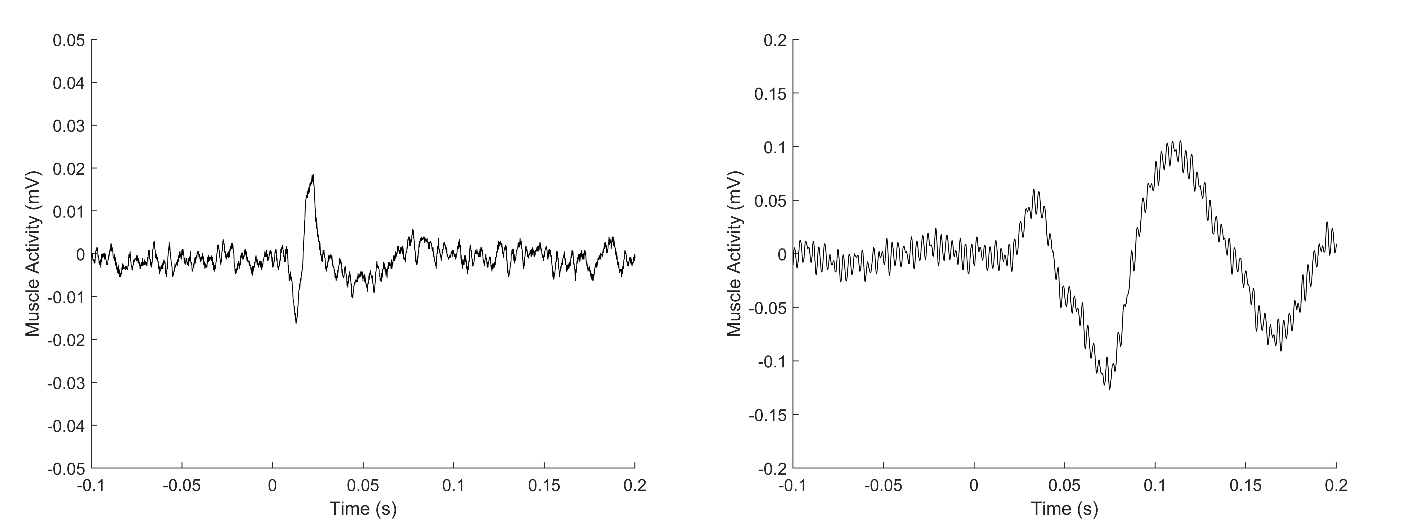


**Figure S2-2.** A typical RRA response from P14 (left) compared to the EMG signal when a movement artifact is present in P11 (right). The movement artifact exhibits a lower frequency, ranging from 1 to 10 Hz, and atypical response amplitude and timing.

# **Supplementary Material S3: Representative Evoked Potentials**


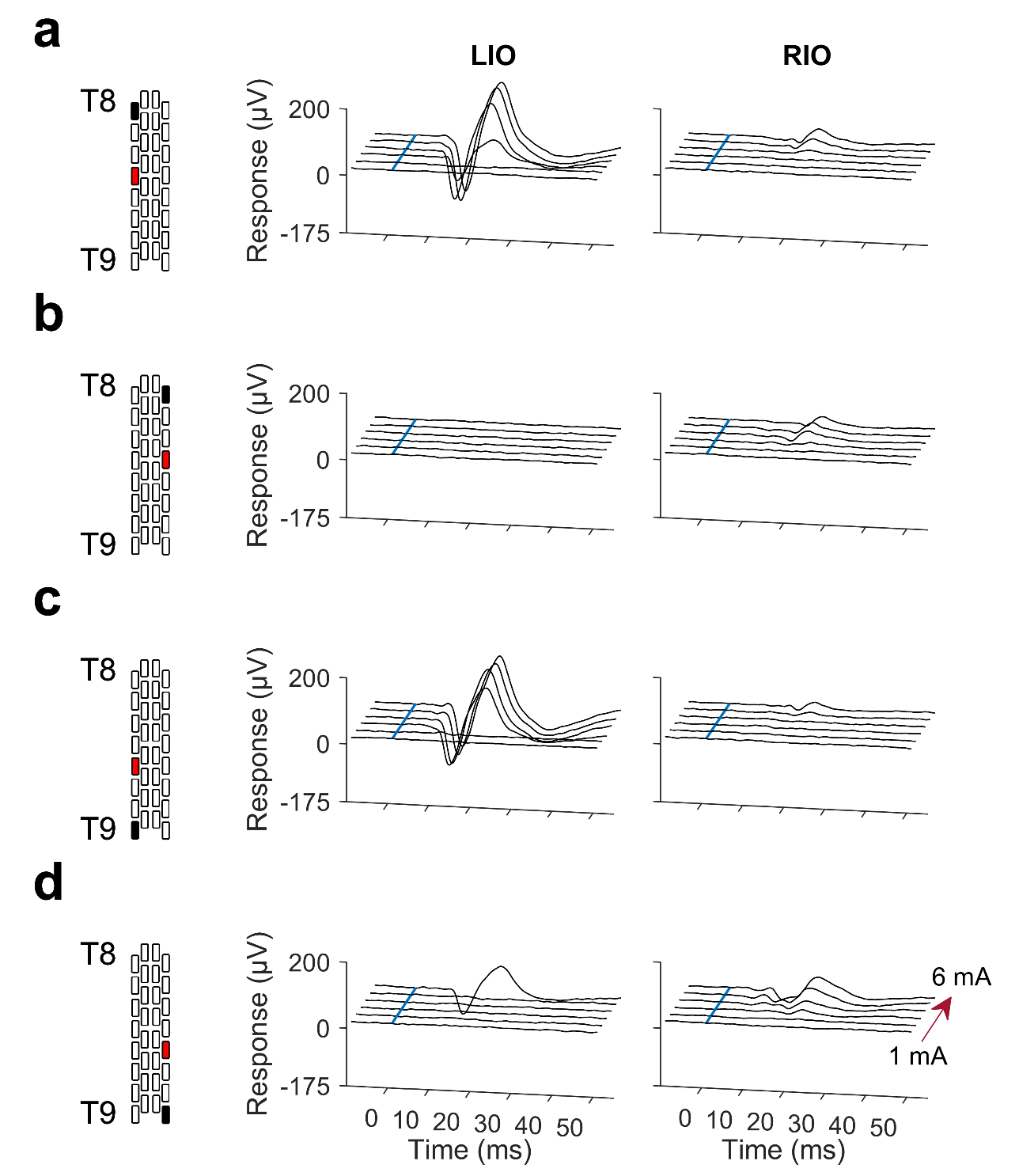


**Figure S3-1.** Representative evoked responses from one participant (P21) for the left and right internal obliques (LIO and RIO), with electrode placement above the T8 and T9 vertebrae. The stimulating cathode is represented in black, and the anode in red. Waveforms, averaged across three trials, are depicted for increasing stimulation amplitudes (1 to 6 mA in 1 mA increments, represented by the red arrow) and when delivering stimulation above: (a) the left T8 vertebra, (b) the right T8 vertebra, (c) the left T9 vertebra, and (d) the right T9 vertebra. Stimulation occurs at zero milliseconds, represented by the blue line.


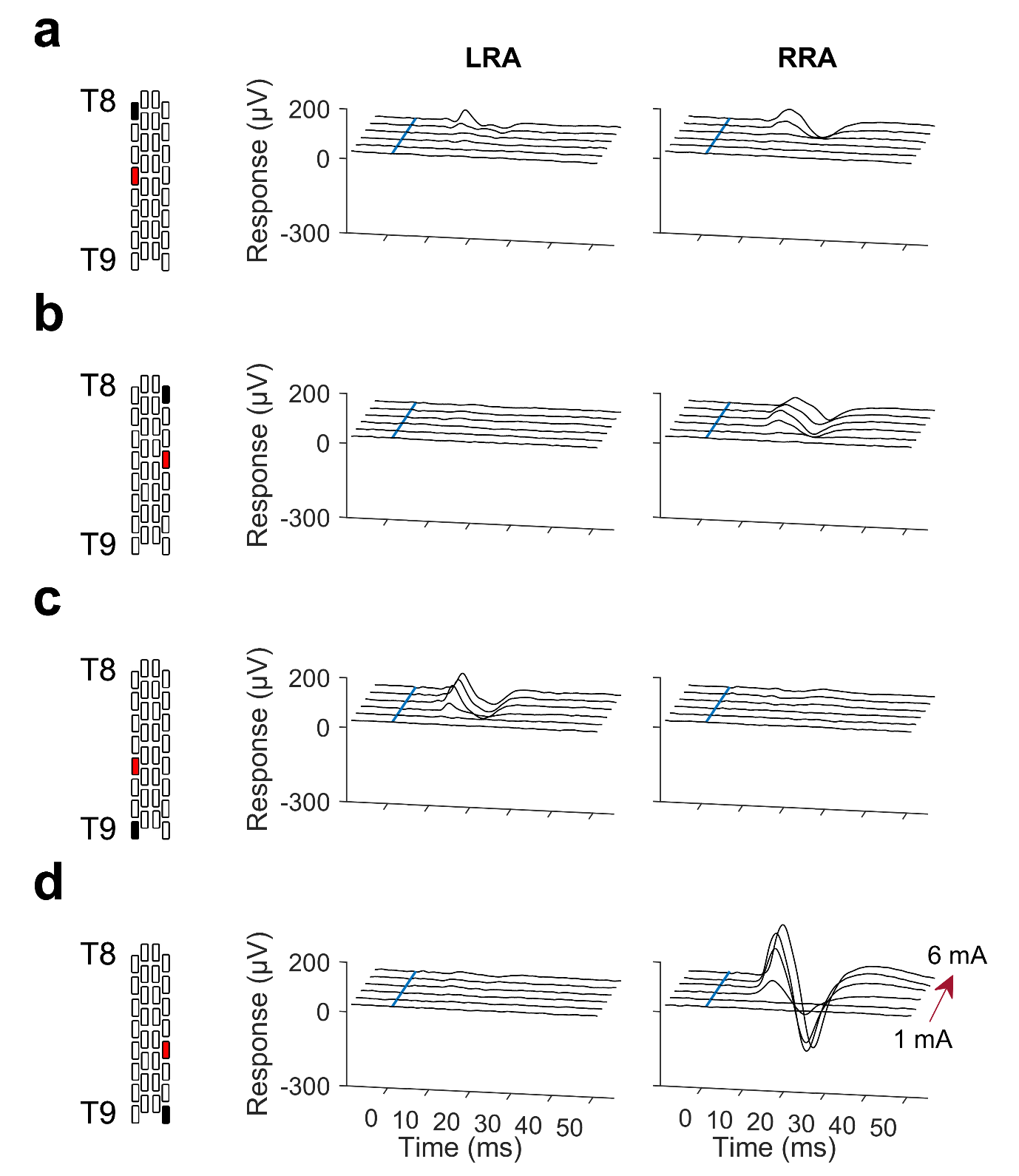


**Figure S3-2.** Representative evoked responses from one participant (P21) for the left and right rectus abdominis (LRA and RRA), with electrode placement above the T8 and T9 vertebrae. The stimulating cathode is represented in black, and the anode in red. Waveforms, averaged across three trials, are depicted for increasing stimulation amplitudes (1 to 6 mA in 1 mA increments, represented by the red arrow) and when delivering stimulation above: (a) the left T8 vertebra, (b) the right T8 vertebra, (c) the left T9 vertebra, and (d) the right T9 vertebra. Stimulation occurs at zero milliseconds, represented by the blue line.


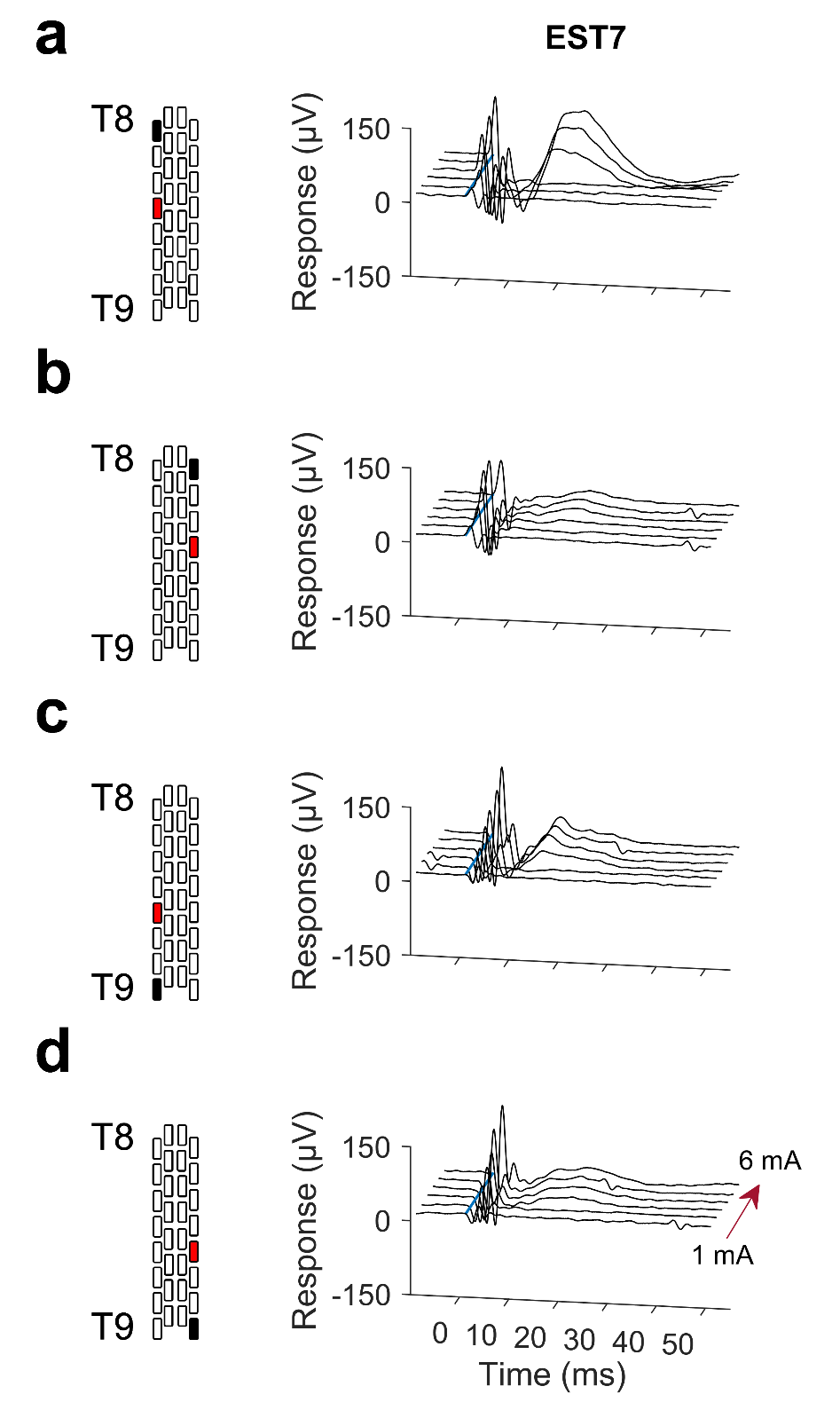


**Figure S3-3.** Representative evoked responses from one participant (P21) for the left erector spinae at the T7 vertebra level (EST7), with electrode placement above the T8 and T9 vertebrae. The stimulating cathode is represented in black, and the anode in red. Waveforms, averaged across three trials, are depicted for increasing stimulation amplitudes (1 to 6 mA in 1 mA increments, represented by the red arrow) and when delivering stimulation above: (a) the left T8 vertebra, (b) the right T8 vertebra, (c) the left T9 vertebra, and (d) the right T9 vertebra. Stimulation occurs at zero milliseconds, represented by the blue line.


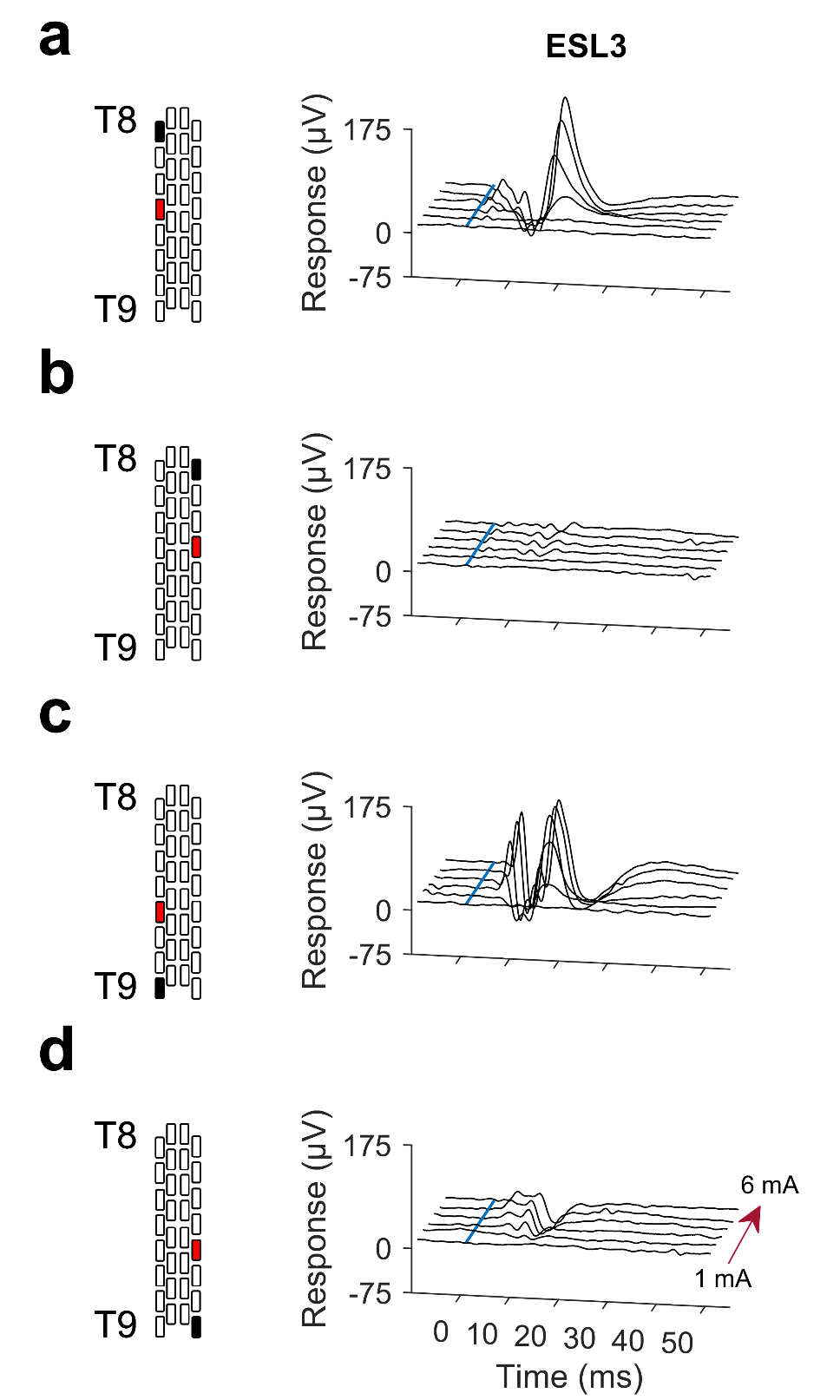


**Figure S3-4.** Representative evoked responses from one participant (P21) for the left erector spinae at the L3 vertebra level (ESL3), with electrode placement above the T8 and T9 vertebrae. The stimulating cathode is represented in black, and the anode in red. Waveforms, averaged across three trials, are depicted for increasing stimulation amplitudes (1 to 6 mA in 1 mA increments, represented by the red arrow) and when delivering stimulation above: (a) the left T8 vertebra, (b) the right T8 vertebra, (c) the left T9 vertebra, and (d) the right T9 vertebra. Stimulation occurs at zero milliseconds, represented by the blue line.

**Supplementary Material S4: Recruitment Curves**

#
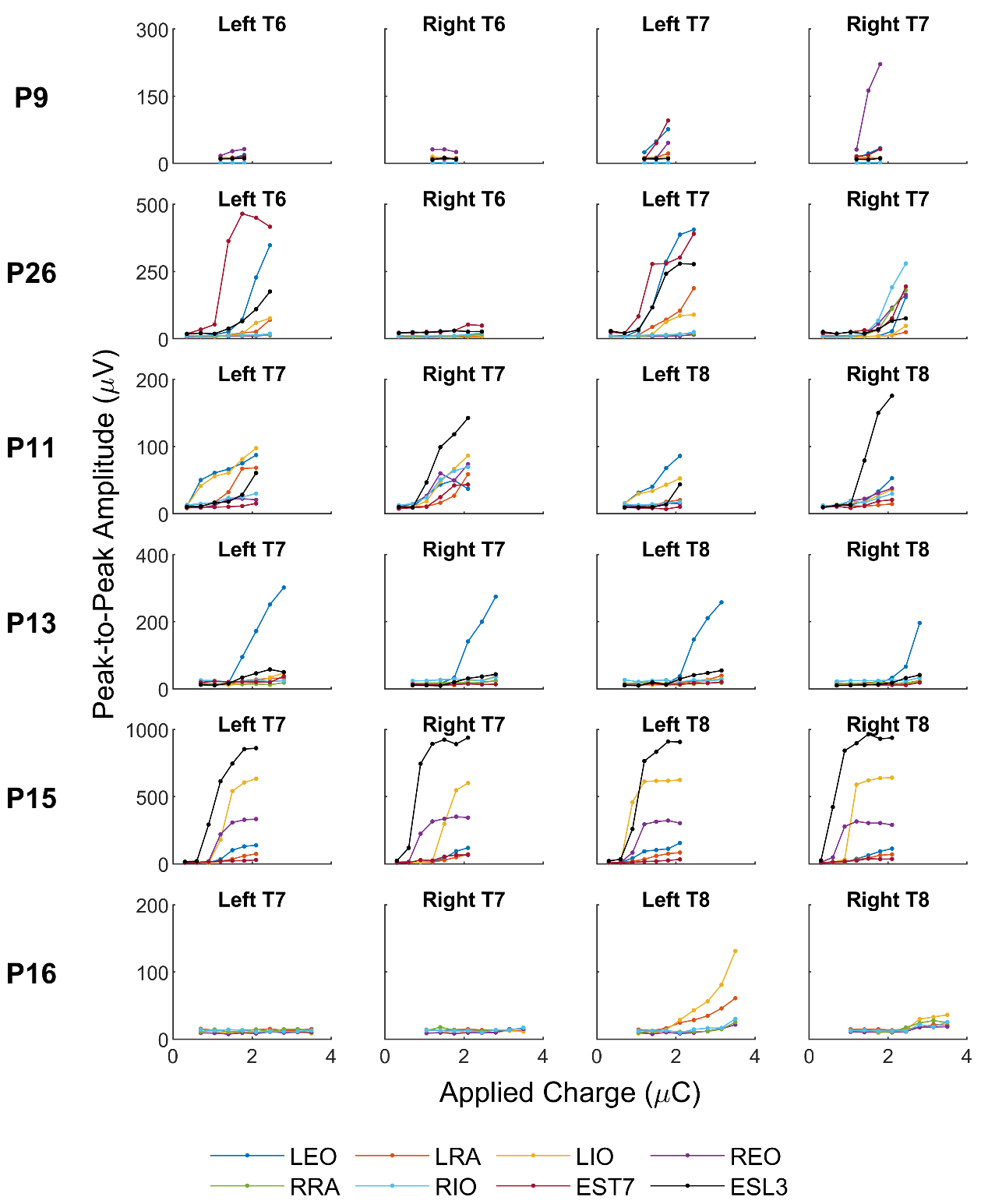


**Figure S4-1.** Representative recruitment curves of muscle responses during stimulation above the T6 and T7 (P9 and P26), and T7 and T8 (P11, P13, P15, and P16) vertebrae. Peak-to-peak amplitudes of the left and right external obliques (LEO and REO), internal obliques (LIO and RIO), rectus abdominis (LRA and RRA), and erector spinae at the T7 and L3 vertebral levels (EST7 and ESL3) are shown in dependence of applied charge. Peak-to-peak amplitude values were averaged across three trials.

**Supplementary Material S5: Onset Latency Analysis for P22**


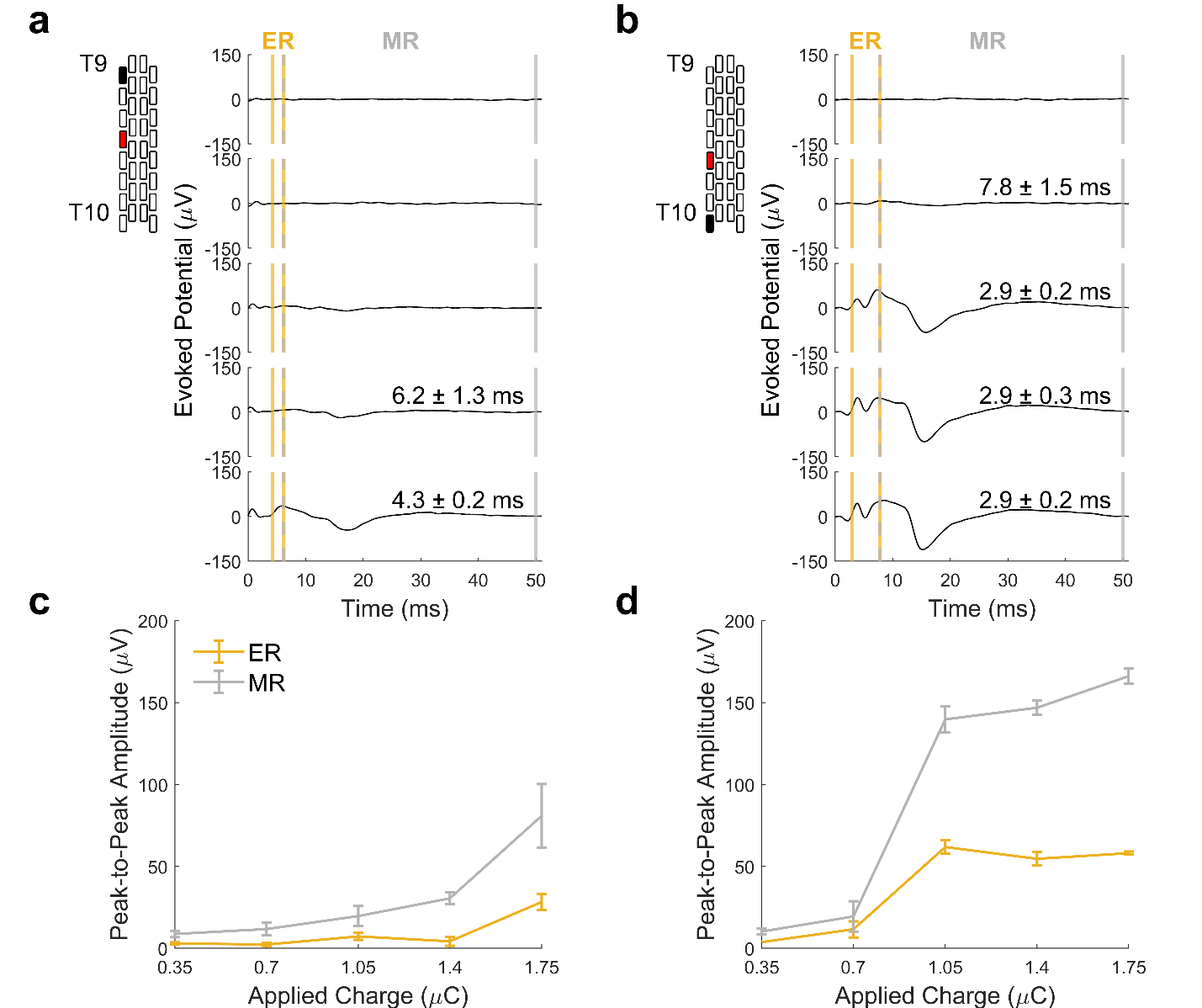


**Figure S5-1**. Onset latency analysis for responses recorded in the ESL3 muscle for P22. Evoked responses with increasing applied charge are shown for ipsilateral stimulation above: (a) the left T9 vertebra, and (b) the left T10 vertebra. Onset latency averaged across three trials (mean ± one standard deviation stated on each waveform) decreased at higher applied charge. The start of the early latency response (ER; vertical solid yellow line) is identified as the onset latency at the highest applied charge for a given electrode configuration. The start of the medium response (MR; vertical dashed yellow and grey line) is identified as the onset latency at the lowest applied charge that evoked a response. The end of MR (vertical solid grey line) is 50 ms. In (c) and (d), the peak-to-peak amplitudes of the ER and MR components are compared when increasing applied charge. All waveforms and outputted metrics were averaged across three trials.

**References**

1. Bobet, J., Masani, K., Popovic, M. R. & Vette, A. H. Kinematics-based prediction of trunk muscle activity in reponse to multi-directional perturbations during sitting. *Med. Eng. Phys.* **58**, 56–63 (2018).

2. Masani, K. *et al.* Postural reactions of the trunk muscles to multi-directional perturbations in sitting. *Clin. Biomech.* **24**, 176–182 (2009).

3. Roberts, B. W. R., Gholibeigian, F., Lewicke, J. & Vette, A. H. Spatial and temporal relation of kinematics and muscle activity during unstable sitting. *J. Electromyogr. Kinesiol.* **52**, (2020).

4. Amrutha, N. & Arul, V. H. A review on noises in EMG signal and its removal. *Int. J. Sci. Res. Publ.* **7**, 23–27 (2017).
